# Influence of nanobody binding on fluorescence emission, mobility, and organization of GFP-tagged proteins

**DOI:** 10.1016/j.isci.2020.101891

**Published:** 2020-12-04

**Authors:** Falk Schneider, Taras Sych, Christian Eggeling, Erdinc Sezgin

**Affiliations:** 1 MRC Human Immunology Unit, MRC Weatherall Institute of Molecular Medicine, University of Oxford, Oxford OX3 9DS, UK; 2 Science for Life Laboratory, Department of Women's and Children's Health, Karolinska Institutet, 171 65 Solna, Sweden; 3 Institute of Applied Optics and Biophysics, Friedrich-Schiller-University Jena, Max-Wien Platz 4, 07743 Jena, Germany; 4 Leibniz Institute of Photonic Technology e.V., Albert-Einstein-Straße 9, 07745 Jena, Germany; 5 Jena Center of Soft Matters, Friedrich-Schiller-University Jena, Philosophenweg 7, 07743 Jena, Germany

**Keywords:** Biochemistry, Biochemistry Methods, Biophysical Chemsitry, Biophysics, Optical Imaging

## Abstract

Advanced fluorescence microscopy studies require specific and monovalent molecular labeling with bright and photostable fluorophores. This necessity led to the widespread use of fluorescently labeled nanobodies against commonly employed fluorescent proteins (FPs). However, very little is known how these nanobodies influence their target molecules. Here, we tested commercially available nanobodies and observed clear changes of the fluorescence properties, mobility and organization of green fluorescent protein (GFP) tagged proteins after labeling with the anti-GFP nanobody. Intriguingly, we did not observe any co-diffusion of fluorescently labeled nanobodies with the GFP-labeled proteins. Our results suggest significant binding of the nanobodies to a non-emissive, likely oligomerized, form of the FPs, promoting disassembly into monomeric form after binding. Our findings have significant implications on the application of nanobodies and GFP labeling for studying dynamic and quantitative protein organization in the plasma membrane of living cells using advanced imaging techniques.

## Highlights

• An anti-GFP nanobody changes GFP fluorescence, brightness, and concentration
• The anti-GFP nanobody increases mobility of GFP-tagged target molecules
• Fluorescently tagged anti-GFP nanobody does not co-diffuse with GFP-tagged proteins
• The anti-GFP nanobody may bind predominantly to dark oligomers of GFP

## Graphical Abstract

**Figure:**
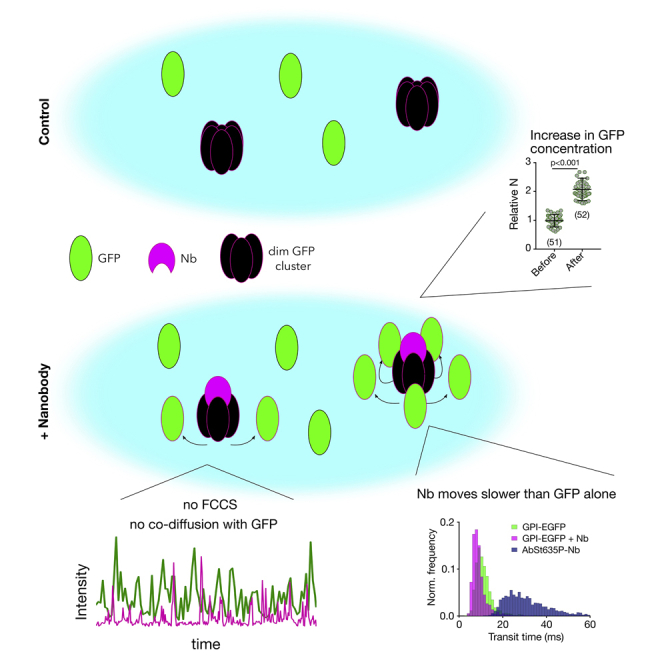

## Introduction

Labeling a protein of interest with an antibody is a well-established procedure in molecular biology. Rather large size and multivalence of antibodies, however, limit their application as labeling agents in imaging approaches. Over the past years, the popularity of antigen-binding fragments of antibodies and single-chain nanobodies derived from camelids or shark antibodies grew vastly (Beghein and Gettemans, 2017; Carrington et al., 2019; Leslie, 2018). Both types of molecules are much smaller than full-length antibodies, yet possess similar binding properties to their target proteins (Harmsen and De Haard, 2007; Sahl et al., 2017). Moreover, they only have a single binding site which prevents cross-linking and artificial clustering (Pereira et al., 2019; Sograte-Idrissi et al., 2020; Stanly et al., 2016). Additionally, the stoichiometric labeling of full length antibodies is challenging, whereas fluorescent labeling of a nanobody with 1:1 (nanobody:dye) ratio is regularly achieved (Gruβmayer et al., 2014). Nanobodies have successfully been raised against various target molecules and used in microscopy (Pleiner et al., 2015, 2018). Some examples for nanobody epitopes include histones (Jullien et al., 2016), viral proteins (Cao et al., 2019), artificial peptides (Braun et al., 2016), clathrin coat components (Traub, 2019), vimentin (Maier et al., 2015) and many more (Aguilar et al., 2019; Mikhaylova et al., 2015). Interestingly, a study using nanobodies targeting synaptic proteins and making use of the nanobodies' smaller size and better penetration capabilities suggested a new pool of synaptic vesicles (Maidorn et al., 2019). The production methods and costs of generating a novel nanobody are higher than the ones for a standard monoclonal antibody; however, the nanobody can subsequently be produced and harvested from bacteria, yeast or mammalian cell culture and even recombinantly tagged (Arbabi Ghahroudi et al., 1997; Beghein and Gettemans, 2017; Pleiner et al., 2018).

The use of nanobodies in microscopy was fueled by the development of a green fluorescent protein (GFP) binding nanobody (Ries et al., 2012). GFP or its derivatives (like the enhanced GFP, EGFP) are attractive targets for super-resolution microscopy as they can be considered the biologist's favorite tag, and a GFP-tagged version of a protein of interest is routinely cloned. However, compared to organic dyes, the brightness and photostability of GFP and its variants are usually worse, limiting its use in some applications (Jensen, 2012; Rankin et al., 2011). Here, the use of anti-GFP nanobodies labeled with, for example, an organic dye with desired chemical or photophysical properties paved the way for a variety of applications (Beghein and Gettemans, 2017; Buser et al., 2018; Fabricius et al., 2018; Farrants et al., 2020; Ries et al., 2012; Sahl et al., 2017) and the development of nanobodies against other fluorescent proteins (FPs) (Platonova et al., 2015). This, in turn, allowed, for example, for multi-color super-resolution imaging with nanobodies (Sograte-Idrissi et al., 2019).

The binding of the anti-GFP nanobody to GFP has been characterized (Kirchhofer et al., 2010; Klamecka et al., 2015; Della Pia and Martinez, 2015), and it has already been noted that the binding of a nanobody to a FP can change the photophysical properties of GFP such as fluorescence brightness, depending on the binding site (Kirchhofer et al., 2010). This influence has been exploited for *in vivo* studies (Llama Tags in fruit fly embryo (Bothma et al., 2018)). General fluorescence properties of GFP have been studied in depth (Conyard et al., 2011; Jung et al., 2005a), and its fluorescence brightness and lifetime, as well as excited- and dark-state populations have been shown to depend on environmental characteristics such as solvent properties (e.g. pH, viscosity), illumination intensity, and wavelength (Ghosh et al., 2017; Jung et al., 2005a; Lippincott-Schwartz and Patterson, 2009; Niwa et al., 1996; Tsien, 1998).

Influences of the nanobody on the functionality of the FP-tagged protein have been indicated before (Küey et al., 2019), and we here present new insights by investigating effects of nanobody binding on the fluorescence emission, organization and mobility of GFP-tagged proteins. Specifically, we used commercially available unlabeled and fluorescently labeled GFP-binding nanobodies (Nb, GFP-Booster) in combination with fluorescence imaging and spectroscopic tools such as fluorescence correlation spectroscopy (FCS) for GFP and EGFP in solution, attached to synthetic membranes, and expressed on the surface of live cells as (E)GFP-tagged glycosylphosphatidylinositol (GPI)-anchored proteins (APs). Our data suggests that the anti-GFP Nb binds a dark oligomeric form of GFP and promotes reorganization by releasing bright monomers.

## Results

### Nanobody binding in solution

**Figure 1:**
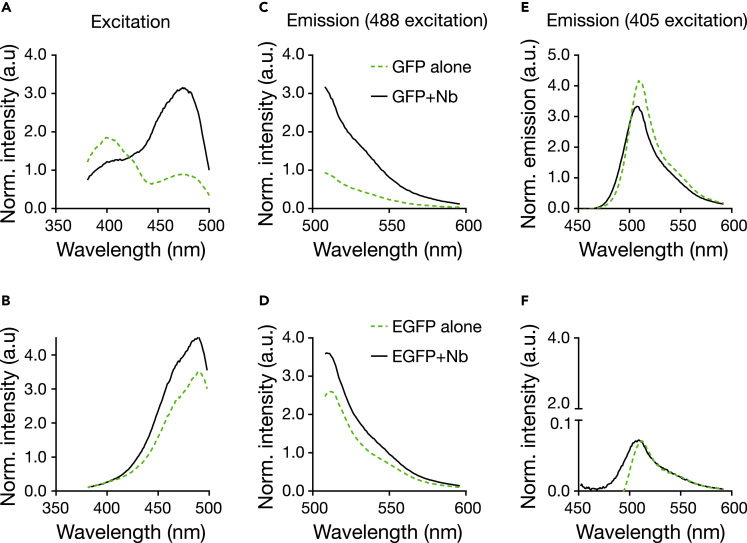
Change in excitation and emission spectra of recombinant GFP and EGFP in solution upon addition of unlabeled Nb (A–F) Excitation spectra for fluorescence detection at 510 - 520 nm (A and B) and emission spectra following 488 nm (C and D) and 405 nm excitation (E and F) of GFP-His (A, C, and E) and EGFP-His (B, D, and F) without Nb (green dashed line) and with excess of unlabeled Nb (solid black line). All spectra are averages of three measurements acquired at 2.5 μg/mL (87 nM) fluorescent protein and 10 μg/mL (720 nM) nanobody in PBS.

We first investigated the basic fluorescence properties of GFP and EGFP before and after addition of unlabeled nanobody (Nb) in solution. Specifically, using a fluorescence spectrometer we investigated changes in total fluorescence intensity and fluorescence spectra for recombinant his-tagged (E)GFP in PBS (pH 7.4, room temperature). Figure 1 shows the respective excitation and emission spectra. As reported before (Tsien, 1998), we found two excitation peaks at around 395 nm and 480 nm for GFP corresponding to the neutral and deprotonated (anionic) state of the fluorochrome, respectively (Chattoraj et al., 1996; Chudakov and Lukyanov, 2003), and one excitation peak at around 480 nm for EGFP (Figures 1A and 1B). The anionic state is usually considered for fluorescence microscopy experiments, using a standard 488 nm laser line for excitation. Due to the requirement for UV excitation, the neutral form of GFP is less used. Interestingly and already previously indicated (Kirchhofer et al., 2010), Nb binding promotes anionic state excitation, revealed by a ≈ 2-fold reduction of the excitation peak at 390 nm and a corresponding ≈ 3-fold increase at 480 nm for GFP (Figure 1A). Similarly but less pronounced, the excitation peak at around 480 nm also increased by 25% for EGFP upon interaction with the Nb (Figure 1B). The Nb binding did not induce any shifts in the emission spectra of GFP or EGFP when excited with either 488 nm (Figures 1C and 1D), or with 405 nm (Figures 1E and 1F), i.e. peak positions of the spectra remained the same. Overall, in solution GFP and EGFP experience a ≈3.5– and ≈1.5–fold increase in total integrated fluorescence emission (510 nm–600 nm) induced by the Nb binding, apparently mainly due to the increase in excitation at around 480 nm. Increase in fluorescence intensity was not markedly dependent on the concentration of the GFP in solution (Figures S1A–S1D). These results are in accordance with *Kirchhofer et al* (Kirchhofer et al., 2010*)*, showing the enhancer nature of the nanobody we use here on our recombinant FPs.

### Nanobody binding on GUV membranes

**Figure 2:**
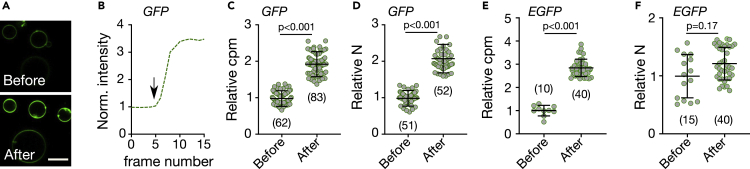
Effect of GFP-nanobody binding on GUV-anchored (E)GFP Data for His-tagged (E)GFP anchored to GUVs (98 mol% DOPC and 2 mol% DGS-NTA) before and after addition of unlabeled Nb as marked. (A) Representative confocal fluorescence microscopy images of the equatorial plane of GUVs decorated with GFP. Scale bar 10 μm. (A–E) (B) Normalized fluorescence intensity over time obtained from subsequently recorded confocal image frames at the equatorial plane of a GFP-tagged GUV, where arrow marks the time of Nb addition. Relative change in (C) molecular fluorescence brightness (cpm) and (D) in number of particles (N) of GFP before and after Nb addition as marked. Relative change in (E) cpm and (D) N of EGFP before and after Nb addition as marked. Values were determined from FCS experiments on individual (E)GFP-tagged GUVs. p-values were determined using the Kolmogorov–Smirnov non-parametric test. Number of data points is indicated on each graph.

In cell biology and microscopy, antibodies and nanobodies are commonly used to investigate the spatial organization of membrane proteins. Therefore, we next tested changes in fluorescence properties of (E)GFP fluorescence at lipid membranes upon binding of unlabeled Nb. We first chose controlled conditions, employing GFP and EGFP attached to synthetic membranes of giant unilamellar vesicles (GUVs, made of dipalmitoylphosphatidylcholine (DOPC) lipid) via a His-tag (using DGS-NTA (1,2-dioleoyl-sn-glycero-3-[(N-(5-amino-1-carboxypentyl)iminodiacetic acid)succinyl])). For both GFP and EGFP, we observed an increase in total fluorescence intensity upon addition of unlabeled Nb, slightly higher (≈2–4 fold) than in solution and with slight differences between GFP and EGFP (4-fold compared to 2.5-fold, respectively) (Figures 2A and 2B). This increase in fluorescence intensity was more prominent at lower GFP concentration but was not concentration-dependent at higher concentrations (Figures S1E and 1F).

To decipher this slight difference in increase in fluorescence intensity further, we tested how the fluorescence emission per individual GFP and EGFP molecule changed upon Nb binding. For this, we determined the molecular fluorescence brightness or fluorescence count rate per molecule, (cpm) derived from FCS (Figures S2A and S2B). FCS reveals the average emitted photons of a fluorophore by measuring photon statistics from multiple transits through the microscope's observation spot. In GUVs, we observed an approximately 2-fold increase in molecular brightness of GFP following Nb addition (Figure 2C), which does not account for the ≈ 4-fold increase in total fluorescence signal intensity (Figure 2B). We therefore also derived the average number of fluorescing molecules, N, from the same FCS experiments (the amplitude of the correlation function, G(0), is inversely correlated to the average number of molecules N). Strikingly, upon Nb addition, we observed an approximately 2-fold increase in N (Figure 2D). For EGFP on GUVs there was also an approximately 2-fold change in molecular brightness cpm (Figure 2E) but in contrast to GFP only a marginal change in N (Figure 2F). As expected, for both GFP and EGFP the increase in N and cpm together account for the overall change in total fluorescence signal intensity (I = cpm × N). It should be noted that N and cpm were derived from fitting the autocorrelation curves and their accuracy depends on the noise in the underlying raw autocorrelation curves. However, several GUVs were measured and the differences in N were significant and cannot be accounted for just by the noise in the raw autocorrelation curves (Figure S2).

While the increase in cpm upon Nb addition may be explained by the change in fluorescence excitation at around 480 nm as determined from the solution experiments (Figures 1A and 1B), the change in N and accordingly in concentration of fluorescing molecules suggests an unexplored enigmatic impact of Nb on the organization of the membrane-bound GFP molecules. We will discuss this and the potential impact on assessing the spatial organization of (E)GFP-tagged proteins in the plasma membrane of living cells in detail throughout the next sections.

### Nanobody binding on live-cell membranes

**Figure 3:**
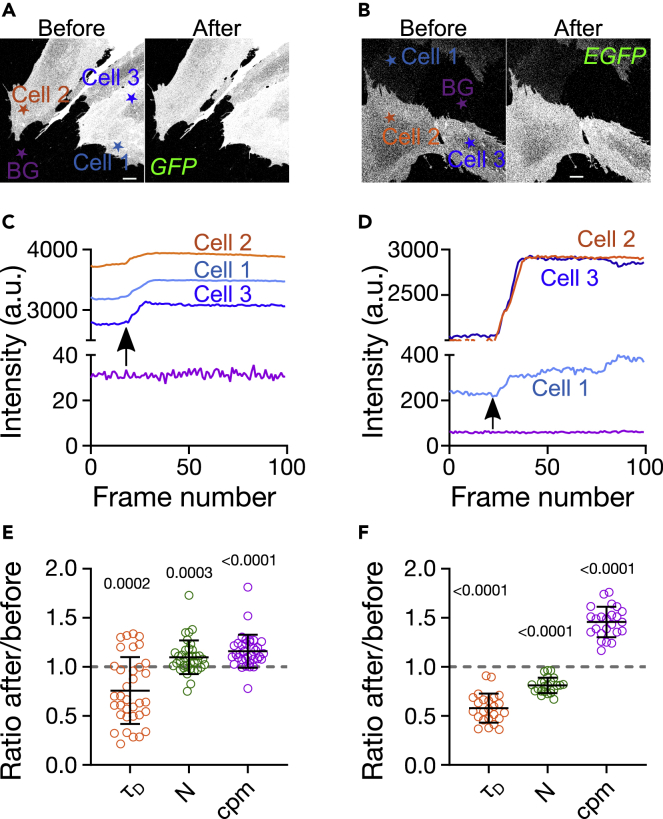
Effect of Nb-binding on (E)GFP in the plasma membrane of live PtK2 cells GPI-anchored proteins GFP-LYPD6 (left panels) and GPI-EGFP (right panels). (A–D) Representative confocal images before and after addition of Nb for (A) GFP-LYPD6 and (B) GPI-EGFP in photon counting mode. Scale bars are 10 μm. Normalized fluorescence intensity traces for (C) GFP-LYPD6 and (D) GPI-EGFP for the cells as indicated in panel a and b, respectively (BG = background). Arrows show the time point when Nb was added. The intensities per frame represent mean values over each cell (see Transparent methods for details). (E and F) Enhancement of fluorescence was ≈1.1-fold for GFP and ≈1.5-fold for EGFP. Change in τ_D_, N and cpm for (E) GFP-LYPD6 and (F) GPI-EGFP upon nanobody addition (values after Nb addition divided by values before). Change in average transit time (τ_D_ i.e. mobility), average fluorescing particle number (i.e. concentration, N), and molecular fluorescence brightness (cpm) upon Nb addition are determined from FCS experiments (one dot = one cell, for each cell 6–9 single FCS measurements were averaged, data was pooled from three different days). The values on top of ratios in e,f indicate p-values obtained from Wilcoxon sign-rank non-parametric tests with hypothetical median values of 1 (ratio of 1 would indicate no change upon Nb addition).

To test the effect of Nb on (E)GFP-tagged membrane protein organization further and in a more physiological setting, we next investigated the influence of Nbs on a GFP- and an EGFP-labeled GPI-AP in the plasma membrane of living cells. Specifically, we expressed GFP-LYPD6 and GPI-EGFP in live PtK2 cells. GFP-LYPD6 is involved in Wnt signaling (Özhan et al., 2013), while GPI-EGFP is simply a lipid-anchored EGFP construct commonly used as probe to study GPI-AP organization (Baumgart et al., 2016; Goswami et al., 2008; Saha et al., 2015; Schneider et al., 2017). We recorded confocal images (Figures 3A and 3B) as well as FCS data (Figures S2C and S2D) to determine the total fluorescence intensity and values of cpm and N. As FCS relies on fluorescence fluctuations, we performed the experiments on cells with relatively low expression levels. For both proteins, we found a modest increase in total fluorescence signal intensity upon Nb binding (Figures 3C and 3D), a slight increase in molecular fluorescence brightness (cpm, ≈1.1-fold for GFP and ≈1.5-fold for EGFP, Figures 3E and 3F), and a distinct variation in average number N of fluorescent molecules in the observation spot, with a slight (≈1.1-fold) increase for GFP and a slight (≈1.2-fold) decrease for EGFP (Figures 3C and 3F). However, especially the determination of the number of particles, N, as well as the related brightness, cpm, was not straightforward on the live-cell membrane due to noise, cellular movements and spatial heterogeneity across the cells (i.e. due to variations in local concentrations across one cell or between different cells, Figures 3A and 3B). Overall, the impact of Nb binding on FP tagged GPI-AP on living cells follows a similar trend as the model membranes with attenuated magnitude (bearing in mind the challenges of live-cell FCS data acquisitions) and suggests reorganization of the lipid-anchored fluorescent proteins upon Nb addition.

### Nanobody effect on molecular mobility

So far, we obtained interesting insights from the stationary thermodynamic information from imaging and FCS (intensity, cpm, and N), but the FCS measurements also allowed us to determine the average mobility of the membrane-APs. Measuring the diffusion dynamics can be performed robustly against local variations in concentration and expression levels and represents a way to study the organization of plasma membrane constituents (Pinkwart et al., 2019; Schneider et al., 2020). From FCS, we obtained the transit time τ_D_, representing the average time it takes a molecule to cross the observation spot, and tested whether it changes upon Nb addition (Figure S2). Intuitively, one may expect a slight decrease in mobility i.e. increase in values of τ_D_ upon addition of Nb due to the increased mass of the complex. Alternatively, one could expect no change at all as the mobility of membrane constituents is overwhelmingly determined by the properties of the membrane anchor (Saffman and Delbrück, 1975; Weiβ et al., 2013). However, interestingly, we observed an increase in mobility after Nb addition for GFP-LYPD6 and GPI-EGFP in the membrane of live PtK2 cells (approximately 1.25-fold and 1.7-fold decrease in values of τ_D_ for GFP and EGFP, respectively; Figures 3E and 3F). From these τ_D_-values and the diameter d = 240 nm of the observation spot (full-width-at-half-maximum), we can estimate values of the diffusion coefficients given the diffusion equation ($D=\frac{d^{2}}{\ln\left(2\right)\cdot 8\cdot \tau_{D}}$) to D = 0.3 μm^2^/s and 0.4 μm^2^/s for GFP-LYPD6 without and with Nb and D = 0.8 μm^2^/s and 1.4 μm^2^/s for GPI-EGFP without and with Nb. Previously reported values for GPI-AP diffusion scatter from 0.3 to 1.0 μm^2^/s (Chojnacki et al., 2017; Eggeling et al., 2009; Huang et al., 2015; Lenne et al., 2006; Schneider et al., 2017; Veerapathiran and Wohland, 2017) where GPI-(E)GFP typically shows faster diffusion than other GPI-anchored probes (such as GPI-ACP or GPI-SNAP).

**Figure 4:**
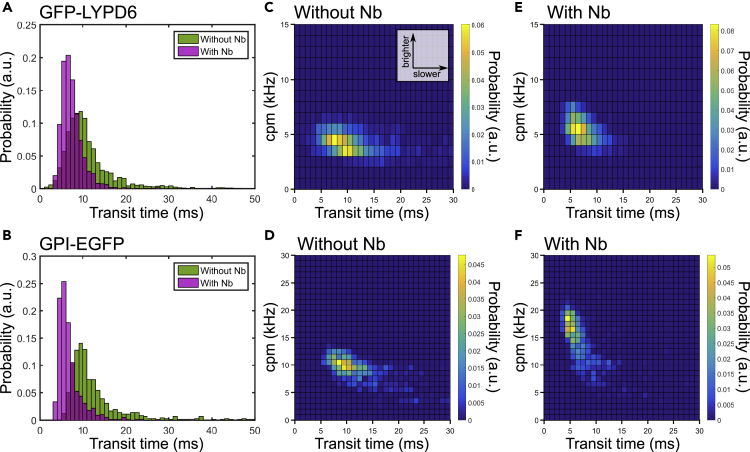
Effect of unlabeled Nb binding on mobility and brightness of GFP-LYPD6 and GPI-EGFP as probed by large sFCS data sets Analysis of diffusion dynamics and molecular brightness (cpm) of GFP-LYPD6 (top panels) and GPI-EGFP (bottom panels) expressed on PtK2 cells and the effect of unlabeled nanobody. (A and B) Transit time histograms for protein without (green) and with (magenta) presence of nanobody. It contains data from >2000 single FCS curves for GFP-LYPD6 and >700 curves for GPI-EGFP from >10 cells each. (C–F) (C and D) Two-dimensional pair value histograms (bivariate histograms) of transit times and cpms for control (without Nb) and with addition of nanobody (E, F) for GFP-LYPD6 (top panels) and GPI-EGFP (bottom panels).

The apparent speed-up upon nanobody binding was puzzling, and to confirm these contradictory findings, we additionally recorded FCS data of GFP-LYPD6 and GPI-EGFP in living cells with higher statistical accuracy to minimize the effect of low sampling in point FCS. Specifically, we performed scanning-FCS (sFCS) measurements, which yield simultaneous FCS data for multiple points along a quickly scanned line. This provides hundreds of values of cpm and τ_D_ with a few measurements, which account for spatial heterogeneity and allow for the determination of average values with very high precision (Schneider et al., 2018; Waithe et al., 2017). The sFCS measurements confirmed the changes in values of τ_D_, i.e. faster diffusion for both GFP-LYPD6 and GPI-EGFP upon Nb binding (Figures 4A and 4B) in line with the point FCS measurements (Figures 3E and 3F). Further, our sFCS data revealed that the increase in mobility (i.e. decrease in transit time τ_D_) was clearly correlated with an increase in brightness, cpm (Figures 4C–4F), i.e. upon Nb addition the population of (E)GFP tagged GPI proteins shifted from a less bright and less mobile to a brighter and more mobile form.

An explanation for these observations could be that the (E)GFP tagged proteins appear to a certain extend in aggregates or homo-oligomers that are (partially) disassembled after Nb binding, leading to an average increase in fluorescent particle number (Figures 2 and 3) and mobility (Figures 3 and 4) without majorly affecting the fluorescence lifetime (Figure S3). GPI-EGFP dimers or GFP oligomers have been reported previously (Aronson et al., 2011; Huang et al., 2015; Jain et al., 2001; Suzuki et al., 2012a) and could be mediated by the FP tag itself (Beutel et al., 2015; Zacharias et al., 2002; Wang et al., 2019). However, since aggregates should in principle be brighter, one would in this case expect a decrease in molecular fluorescence brightness (cpm) upon aggregate disassembly. Our opposite observation indicates that the aggregates might be darker (or significantly dimmer), e.g. due to self-quenching processes (Ge et al., 2017; Jung et al., 2005b), and their fraction is rather low after disassembly. This is clearly illustrated by the bivariate histograms of cpm and τ_D_ (Figures 4C–4F); the fraction of dimmer and slower molecules (“tail” of the distribution in Figures 4C and 4D) is notably reduced in the presence of nanobody (Figures 4E and 4F). If this is indeed the case, it is essential to know whether the supposedly monovalent Nb binds to both oligomers and monomers with different affinity or selectively to one pool.

### Diffusion of labeled nanobodies

**Figure 5:**
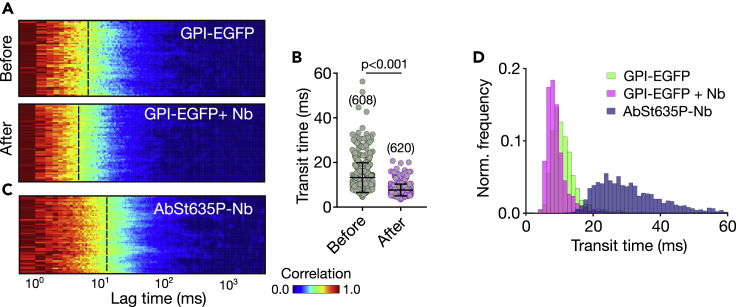
Diffusion of labeled Nb (AbStar635P-Nb) on GPI-EGFP expressing PtK2 Cells (A) Representative normalized sFCS autocorrelation carpets for (A) GPI-EGFP before and after addition of AbStar635P-Nb (x-axis: correlation lag time, y-axis: line pixels (space), color scale: normalized correlation from zero (blue) to one (red)), revealing a shift of average transit time (yellow region, average transit time highlighted by the dashed line) toward shorter times after addition of labeled Nb. (B) τ_D_ values for GPI-EGFP before and after AbStar635P-Nb addition including mean values and standard deviations. (C) Normalized autocorrelation carpet for AbStar635P-Nb bound to PtK2 cells expressing GPI-EGFP. (D) Histogram of τ_D_ for GPI-EGFP (with and without Nb) and AbStar635P-Nb. The p-value given in panel B was calculated using the Kolmogorov–Smirnov non-parametric test.

To address what species is bound by the nanobody, we measured sFCS and FCS with fluorescently labeled Nb and GFP-LYPD6 or GPI-EGFP at the plasma membrane of live PtK2 cells. Specifically, we employed Nbs tagged with the red-emitting dye Abberior Star 635P (AbStar635P-Nb), whose fluorescence emission was clearly distinguishable from that of the FPs and detected on a separate detector. First, using confocal imaging we confirmed that the AbStar635-Nb bound only to the surface transfected cells and not to those without e.g. GPI-EGFP, i.e. AbStar635-Nb specifically interacted with the EGFP on the membrane only (Figure S4). Next, we also found an increase in mobility (i.e. decrease in average transit time τ_D_) and increase in brightness cpm of the EGFP tagged proteins upon AbStar635-Nb binding, i.e. the label did not influence this effect (Figures 5A and 5B and S5). Interestingly, simultaneously recorded sFCS data for AbStar635P-Nb and GPI-EGFP (Figures 5C and 5D) revealed a profoundly slower diffusion for AbStar635P-Nb compared to the EGFP-tagged proteins. We observed an average transit time of τ_D_ = 28.3 ms ± 9.4 (D = 0.5 ± 0.17 μm^2^/s) for AbStar635P-Nb and τ_D_ = 10.3 ms ± 1.0 (D = 1.2 ± 0.12 μm^2^/s) for GPI-EGFP (Figures 5A–5D). This is an obvious contradiction, as the Nbs should be bound directly to the surface proteins (GPI-EGFP) but moved significantly slower than the protein itself. A possible explanation for this contradiction extends our previous hypothesis and points to the existence of at least two pools of EGFP on the cell surface; a darker oligomeric form that diffuses slowly to which the Nb preferentially binds. Nb binding to this pool drives the partial displacement of brighter and faster moving monomers, to which Nb does not bind efficiently.

### Missing co-diffusion of (E)GFP and nanobodies

**Figure 6:**
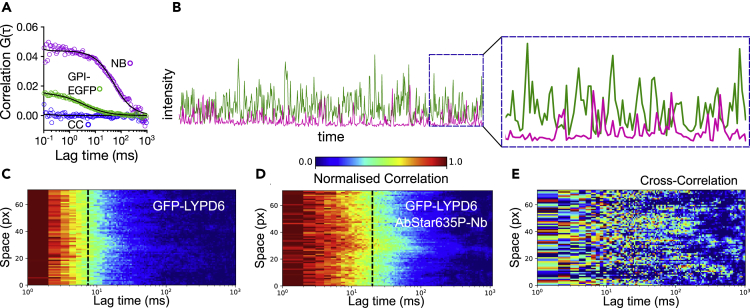
Missing co-diffusion of labeled Nb and (E)GFP-tagged surface proteins PtK2 cells expressing GPI-EGFP or GFP-LYPD6 were treated with labeled Nb (AbStar635P-Nb) and FCCS data acquired. Positive cross correlation (CC) indicates interaction, i.e. co-diffusion. (A) point FCCS of GPI-EGFP and Nb. Autocorrelation EGFP is green, autocorrelation of AbberiorStar635P-labeled Nb is magenta and cross correlation (CC) is blue. (B) Representative dual-color intensity trace showing that the detection events for EGFP and AbStar635P-Nb rarely overlap in time. (C–E) Scanning FCCS data of GFP-LYPD6 expressed on the surface of PtK2 cells and Nb, with representative normalized auto-correlation data for (C) GFP-LYPD6, (D) AbStar635P-Nb and (E) normalized cross-correlation data of these two. The dashed black line indicates the average transit times. The temporal cross-correlation of these two dataset does not show any positive cross-correlation, i.e. no co-diffusion.

Although Figure S4 shows that Nb binds exclusively to the GFP-positive cells, there is still the possibility that Nb interacts non-specifically with the membrane after binding to GFP on the surface which could lead to a slowdown in Nb diffusion. In this scenario, we would observe slowed-down co-diffusion of Nb with GFP due to the interaction of Nb with the membrane. To investigate this, we applied fluorescence cross correlation spectroscopy (FCCS) (Schwille et al., 1997). Based on the principle of FCS, FCCS takes information from the temporal cross-correlation function of two simultaneously recorded fluorescence signal time traces of two distinctively labeled (e.g. green and red fluorescence, respectively) diffusing molecules to determine the degree of co-diffusion or interaction of the two molecules. Only when molecules show co-diffusion or interaction, the amplitude of the cross-correlation curve is larger than zero. An FCCS amplitude of zero indicates the absence of co-diffusion of the two fluorescing molecules (Bacia and Schwille, 2007). Therefore, the AbStar635P-Nb binding to fluorescently tagged GPI-APs should be a perfect sample for FCCS analysis, since every (red-emitting) Nb molecule should be bound to a (green-emitting) (E)GFP, yielding in theory a perfect cross-correlation between the red and green fluorescence signals. Performance of our FCCS experiments was validated through a positive control (red-labeled peptide binding specifically to membrane-embedded green-emitting cholesterol analog, Figure S6), showing a large non-zero FCCS amplitude, confirming near-perfect co-diffusion. Strikingly, we did not observe any notable cross-correlation and therefore no co-diffusion between AbStar635P-Nb and (E)GFP-tagged molecules anchored to the plasma membrane, as shown for GPI-EGFP in Figure 6A. The absence of co-diffusion is also illustrated by the dual-color intensity time trace (Figure 6B) demonstrating only very rare detection events with signal from both channels, i.e. EGFP and AbStar635P-Nb independently crossed the observation spot. Following the same strategy as before, we used sFCS to confirm these findings for GFP-LYPD6, employing higher statistical throughput and spatial sampling. The auto-correlation carpets (Figures 6C and 6D) reveal the same slower diffusion of the bound AbStar635P-Nb compared to the binding partner GFP-LYPD6, as was the case for the experiments with GPI-EGFP (Figures 5A and 5C). Similarly, the scanning cross-correlation data of AbStar635P-Nb and GFP-LYPD6, which showed noise but no notable signal (in contrast to a positive control, Figure S7), confirmed the complete absence of co-diffusion of fluorescing binding partners (Figure 6E). We can conclude that in our experiments the Nbs do not bind the bright and fast diffusing (E)GFP-tagged proteins (supposedly monomers) but predominantly to a dark and slowly diffusing entity (supposedly oligomers).

We did not observe positive cross-correlation in simple one-phase (98 mol% DOPC and 2 mol% DGS-Ni-NTA) model membranes decorated with bare His-tagged GFP (i.e., no other proteins attached to GFP), showing that the proteins attached to GFP cannot be the reason behind lack of co-diffusion (Figure S8). This also shows that there is not a major contribution of live cell plasma membrane complexity to the observed effects. In addition, we performed the same FCCS experiments in solution and similarly, we do not see any cross-correlation between recombinant GFP and fluorescent Nb (Figure S8). This confirms that the membrane environment or the protein labeled with GFP do not contribute to the lack of co-diffusion. It is rather a combined effect of Nb and FP. A lack of co-diffusion even at very small concentration of GFP in the system (Figure S8) shows that GFP inherently has two pools (bright and dark) independent of concentration.

Other reasons for the absence of cross-correlation could be (*i)* very fast binding kinetics (i.e. on- and off-rates) of the Nb-(E)GFP interaction or *ii)* too low binding leaving too many unbound (E)GFP molecules. However, our data do not support these scenarios. (1) We determined off-rates, k_off_, for the Nb-GFP binding using surface plasmon resonance (SPR) experiments (of GFP binding to surface-immobilized (labeled and unlabeled) Nbs), resulting in k_off_ ≈ 5 × 10^−4^ s^−1^ (Figure S9). Consequently, the Nb-(E)GFP complex is stable for about 30 min, which is in agreement with previous data (Della Pia and Martinez, 2015). During the 1-50 milliseconds long transit through the observation spot, the Nb-(E)GFP complex should be intact. This data rules out fast kinetics. (2) We also recorded FCCS data in large excess of AbStar635-Nb, saturating GFP binding. However, we still did not observe any cross-correlation (Figure S10). This data rules out the domination of unbound (E)GFP particles.

We tested whether the lack of cross-correlation signal could be dye-specific for AbStar635P, but cross-correlation was also absent when performing the same sFCCS experiments as before with nanobody labeled with the dye Atto594 (Figure S11).

Another possible explanation for the lack of co-diffusion might be an extremely efficient (close to 100%) energy transfer (Sun et al., 2011) between the FPs and the AbStar635-Nb. Such a Förster resonance energy transfer (FRET) would render the Nb-bound FPs very dim and therefore hardly visible for FCCS analysis (FCS-based experiments require rather large fluorescence brightness (Saffarian and Elson, 2003; Schneider et al., 2020)). Several observations oppose this scenario: (i) Close to 100% FRET efficiency should lead to a huge decrease in the number N of visible donor (E)GFP molecules and thus a decrease of (E)GFP fluorescence intensity with nanobody binding, which is not the case (Figures 1, 2, and S12). (ii) A large energy transfer generally leads to a vast decrease in the fluorescence lifetime of the FRET donor, in this case (E)GFP. We therefore measured and compared values of the fluorescence lifetime for GFP and EGFP with and without binding to unlabeled and AbStar635P-labeled Nb. There was a small reduction in fluorescence lifetime for GFP (≈3 ns to ≈2.2 ns) and EGFP (≈2.6 ns to ≈2.1 ns) in solution (Figure S13), indicating only a minor influence by FRET and not explaining the complete lack of cross-correlating fluorescence signal in cells, in GUVs and in solution (Figure S8). Some FRET may explain though the lower increase in fluorescence intensity upon labeled nanobody binding to the recombinant proteins in solution (Figure S14) compared to unlabeled Nb (Figure 1). iii) The molecular fluorescence brightness cpm of the donor EGFP molecules should go down after addition of labeled compared to unlabeled Nbs. As highlighted before, we however see an increase in cpm values independent of labeled or unlabeled Nb (Figures 4C–4F and S5).

## Discussion

The use of Nbs presents a versatile new route for detection and manipulation of proteins in biology and especially microscopy (Beghein and Gettemans, 2017). Their small size, monovalancy, the ability to label them stoichiometrically, and their recombinant or even *in vivo* production make them an attractive tool (Bothma et al., 2018; Gruβmayer et al., 2014; Sograte-Idrissi et al., 2020). The GFP-binding Nbs along with Nbs against other FPs enable conveniently to perform super-resolution microscopy on the tagged protein of interest (Platonova et al., 2015; Ries et al., 2012; Sograte-Idrissi et al., 2019). A modulation of the GFP's spectral properties by interaction with Nbs has been shown and exploited before (Bothma et al., 2018; Kirchhofer et al., 2010); however, the Nb has not been implicated in the reorganization of the FP-tagged protein.

In this study, we used imaging and spectroscopic techniques to investigate changes in the dynamic organization of (E)GFP in solution, in model membranes, and in living cells upon binding of labeled and unlabeled anti-GFP nanobody. Overall, our data show that (i) Nb increases the apparent number of GFP molecules on GUVs and cells, (ii) Nb binding to FPs on living cells increases the mobility of GPI-anchored EGFP or GFP, (iii) the Nb diffuses slowly compared to the GFP on living cells, which means that they do not co-diffuse, and (iv) the Nb binding modulates photophysical properties of the FPs. These findings may suggest that the Nbs bind predominantly to an already existing dark pool of the (E)GFP, which might be a slow-moving, higher order complex (or large oligomers of partially misfolded FP). Lack of co-diffusion suggests that this complex is not strongly fluorescent (e.g., due to self-quenching) but still primarily recognized by the Nb. Fluorescence modulation upon aggregation or mis-folding of FPs and their chromophores has been described before (Camacho et al., 2018; Ge et al., 2017; Jung et al., 2005b; Kruitwagen et al., 2015; Stepanenko et al., 2008). Upon binding, Nb could partially disassemble the dark complex and release a few molecules from the complex that become fluorescent but are not bound to a Nb. The observed effects take place in solution as well as in model membranes. These systems are devoid of active protein organization, i.e., in thermodynamically equilibrium. No cross-correlation between Nb and GFP as well as increase in number of molecules in these systems suggest that the change in organization upon nanobody binding is due to a darker pool of the FP independent of cellular environment. The dark pool and bright pool of the FP might not be in constant exchange as the observed effects seem not to be dependent on the concentration. Thus, the nanobody addition might trigger an irreversible change such as refolding and release of misfolded protein from within the dark clusters. Moreover, the effects were present even in very low concentrations of GFP in the system, suggesting the presence of inherently different pools of GFP when it is both in 3D environment (isolated form in solution) and in 2D environment (attached to membranes).

Overall, our results show that the Nb binding could influence the organization of the GFP-tagged proteins at the membrane of living cells. It has been reported before that Nb binding could perturb protein function (Küey et al., 2019). Consequently, when performing conventional or super-resolved imaging using anti-GFP nanobodies to label (E)GFP conjugated proteins, the measurements need to be interpreted with great care, especially on live cells and when quantitative data on dynamics are derived.

### Limitations of the study

Here, we only showed the effect of one specific anti-GFP nanobody (GFP-booster from Chromotek) and we also acknowledge that we only tested one GFP and one EGFP variant expressed on the surface of the living cells (see Transparent methods for protein sequences). Many derivatives of GFP exist that counteract FP-mediated oligomerization especially when passing through oxidative environments (Aronson et al., 2011; Zacharias et al., 2002; Suzuki et al., 2012b; Wang et al., 2019). An interesting approach could be to add a short tag to the FP to allow detection with another nanobody (Braun et al., 2016).

Relying on fluorescence does not readily allow to quantify the dark fraction of the molecules. Using label-free techniques such iSCAT microscopy in combination with supported lipid bilayers and the lipid anchored GFPs could provide additional insights (Reina et al., 2018; De Wit et al., 2015). Similarly, our major readout is the fluctuation analysis (FCS, FCCS and sFC(C)S). These techniques are robust technologies for this type of applications but do have some pitfalls. For example, as mentioned above, the molecular brightness is the major determinant for the FCS data quality and thus very dim molecules are dominated by bright molecules. Therefore, when there are two pools (bright and dim, with the dim pool being minor in concentration), the two components may not be represented accurately in the extracted fit data as the brighter component dominates the signal. We always fitted the data with the simplest model possible which is considered as the good fitting practise (not using additional components if the data is fitted well with one component). For example, our sFCS curves were in all cases well described by a single component model without anomalous sub-diffusion (Figure S15). Of course, that may oversee a minor pool showing different dynamics. As a remedy we used the combination of point and scanning FCS to cover a wider range of temporal sensitivity.

We refrain ourselves from generalizing the described effect to all nanobodies and other GFP variants and just emphasize on the possible influence of the tag on the protein of interest's organization. We do not discourage the use of (E)GFP-tags and nanobodies but want to draw attention to possibly misleading artifacts. Evaluation of other nanobodies and FPs are ongoing efforts in the lab. However, recently, by using SNAP-25 and Syntaxin 1A nanobodies, a previously undetected pool of synaptic population was found in the cells (Maidorn et al., 2019), which was attributed to nanobodies' ability to reveal different organization patterns. Therefore, there is a possibility that the effect of nanobodies could be more general. Moreover, here we only assess the effect of nanobodies against His-tagged (E)GFP proteins in model membranes and in solution or (E)GFP-labeled GPI-APs, and the observed effects are surely a combination of nanobodies and FPs. Yet, these are all widely used and the effect will likely be similar on other proteins (e.g. transmembrane or cytoplasmic) labeled with GFP or its derivatives as the modulating interactions are between the FP and the nanobody.

### Resource availability

#### Lead contact

Any requests for resources or further information should be directed to the lead contact Erdinc Sezgin (erdinc.sezgin@ki.se).

#### Materials availability

This study did not generate new unique reagents. Materials are available from the authors on request.

#### Data and code availability

This study did not generate large-scale data sets or novel unique analysis code. All data are described in the main text and all analysis methods in the Supplemental information.

## Methods

All methods can be found in the accompanying Transparent methods supplemental file.

## Supplemental information

Document S1. Transparent methods and Figures S1–S17

## Footnotes

Supplemental Information can be found online at https://doi.org/10.1016/j.isci.2020.101891.

## References

[bib1] Aguilar G., Matsuda S., Vigano M.A., Affolter M. (2019). Using nanobodies to study protein function in developing organisms. Antibodies.

[bib2] Arbabi Ghahroudi M., Desmyter A., Wyns L., Hamers R., Muyldermans S. (1997). Selection and identification of single domain antibody fragments from camel heavy-chain antibodies. FEBS Lett..

[bib3] Aronson D.E., Costantini L.M., Snapp E.L. (2011). Superfolder GFP is fluorescent in oxidizing environments when targeted via the sec translocon. Traffic.

[bib4] Bacia K., Schwille P. (2007). Practical guidelines for dual-color fluorescence cross-correlation spectroscopy. Nat. Protoc..

[bib5] Baumgart F., Arnold A.M., Leskovar K., Staszek K., Fölser M., Weghuber J., Stockinger H., Schütz G.J. (2016). Varying label density allows artifact-free analysis of membrane-protein nanoclusters. Nat. Methods.

[bib7] Beghein E., Gettemans J. (2017). Nanobody technology: a versatile toolkit for microscopic imaging, protein–protein interaction analysis, and protein function exploration. Front. Immunol..

[bib8] Beutel O., Roder F., Birkholz O., Rickert C., Steinhoff H.J., Grzybek M., Coskun Ü., Piehler J. (2015). Two-dimensional trap for ultrasensitive quantification of transient protein interactions. ACS Nano.

[bib9] Bothma J.P., Norstad M.R., Alamos S., Garcia H.G. (2018). LlamaTags: a versatile tool to image transcription factor dynamics in live embryos. Cell.

[bib10] Braun M.B., Traenkle B., Koch P.A., Emele F., Weiss F., Poetz O., Stehle T., Rothbauer U. (2016). Peptides in headlock - a novel high-affinity and versatile peptide-binding nanobody for proteomics and microscopy. Sci. Rep..

[bib11] Buser D.P., Schleicher K.D., Prescianotto-Baschong C., Spiess M. (2018). A versatile nanobody-based toolkit to analyze retrograde transport from the cell surface. Proc. Natl. Acad. Sci. U S A.

[bib12] Camacho R., Täuber D., Hansen C., Shi J., Bousset L., Melki R., Li J.Y., Scheblykin I.G. (2018). 2D polarization imaging as a low-cost fluorescence method to detect α-synuclein aggregation ex vivo in models of Parkinson’s disease. Commun. Biol..

[bib13] Cao J., Zhong N., Wang G., Wang M., Zhang B., Fu B., Wang Y., Zhang T., Zhang Y., Yang K. (2019). Nanobody-based sandwich reporter system for living cell sensing influenza A virus infection. Sci. Rep..

[bib14] Carrington G., Tomlinson D., Peckham M. (2019). Exploiting nanobodies and Affimers for superresolution imaging in light microscopy. Mol. Biol. Cell.

[bib15] Chattoraj M., King B.A., Bublitz G.U., Boxer S.G. (1996). Ultra-fast excited state dynamics in green fluorescent protein: multiple states and proton transfer. Proc. Natl. Acad. Sci. U S A.

[bib16] Chojnacki J., Waithe D., Carravilla P., Huarte N., Galiani S., Enderlein J., Eggeling C. (2017). Envelope glycoprotein mobility on HIV-1 particles depends on the virus maturation state. Nat. Commun..

[bib17] Chudakov D.M., Lukyanov K.A. (2003). Use of green fluorescent protein (GFP) and its homologs for in vivo protein motility studies. Biochem.

[bib18] Conyard J., Kondo M., Heisler I.A., Jones G., Baldridge A., Tolbert L.M., Solntsev K.M., Meech S.R. (2011). Chemically modulating the photophysics of the GFP chromophore. J. Phys. Chem. B.

[bib19] Eggeling C., Ringemann C., Medda R., Schwarzmann G., Sandhoff K., Polyakova S., Belov V.N., Hein B., von Middendorff C., Schönle A. (2009). Direct observation of the nanoscale dynamics of membrane lipids in a living cell. Nature.

[bib20] Fabricius V., Lefèbre J., Geertsema H., Marino S.F., Ewers H. (2018). Rapid and efficient C-terminal labeling of nanobodies for DNA-PAINT. J. Phys. D. Appl. Phys..

[bib21] Farrants H., Tarnawski M., Müller T.G., Otsuka S., Hiblot J., Koch B., Kueblbeck M., Kräusslich H.-G., Ellenberg J., Johnsson K. (2020). Chemogenetic control of nanobodies. Nat. Methods.

[bib22] Ge S., Deng H., Su Y., Zhu X. (2017). Emission enhancement of GFP chromophore in aggregated state via combination of self-restricted effect and supramolecular host–guest complexation. RSC Adv..

[bib23] Ghosh A., Isbaner S., Veiga Gutierrez M., Gregor I., Enderlein J., Karedla N. (2017). Quantifying microsecond transition times using fluorescence lifetime correlation spectroscopy. J. Phys. Chem. Lett..

[bib24] Goswami D., Gowrishankar K., Bilgrami S., Ghosh S., Raghupathy R., Chadda R., Vishwakarma R., Rao M., Mayor S. (2008). Nanoclusters of GPI-anchored proteins are formed by cortical actin-driven activity. Cell.

[bib25] Grußmayer K.S., Kurz A., Herten D.P. (2014). Single-molecule studies on the label number distribution of fluorescent markers. ChemPhysChem.

[bib26] Harmsen M.M., De Haard H.J. (2007). Properties, production, and applications of camelid single-domain antibody fragments. Appl. Microbiol. Biotechnol..

[bib27] Huang H., Simsek M.F., Jin W., Pralle A. (2015). Effect of receptor dimerization on membrane lipid raft structure continuously quantified on single cells by camera based fluorescence correlation spectroscopy. PLoS One.

[bib29] Jain R.K., Joyce P.B.M., Molinete M., Halban P.A., Gorr S.-U. (2001). Oligomerization of green fluorescent protein in the secretory pathway of endocrine cells. Biochem. J..

[bib31] Jensen E.C. (2012). Use of fluorescent probes: their effect on cell biology and limitations. Anat. Rec. Adv. Integr. Anat. Evol. Biol..

[bib32] Jullien D., Vignard J., Fedor Y., Béry N., Olichon A., Crozatier M., Erard M., Cassard H., Ducommun B., Salles B. (2016). Chromatibody, a novel non-invasive molecular tool to explore and manipulate chromatin in living cells. J. Cell Sci..

[bib33] Jung G., Wiehler J., Zumbusch A. (2005). The photophysics of green fluorescent protein: influence of the key amino acids at positions 65, 203, and 222. Biophys. J..

[bib34] Jung K., Park J., Maeng P.J., Kim H. (2005). Fluorescence quenching of green fluorescent protein during denaturation by guanidine. Bull. Korean Chem. Soc..

[bib35] Kirchhofer A., Helma J., Schmidthals K., Frauer C., Cui S., Karcher A., Pellis M., Muyldermans S., Casas-Delucchi C.S., Cardoso M.C. (2010). Modulation of protein properties in living cells using nanobodies. Nat. Struct. Mol. Biol..

[bib36] Klamecka K., Severin P.M., Milles L.F., Gaub H.E., Leonhardt H. (2015). Energy profile of nanobody–GFP complex under force. Phys. Biol..

[bib37] Kruitwagen T., Denoth-Lippuner A., Wilkins B.J., Neumann H., Barral Y. (2015). Axial contraction and short-range compaction of chromatin synergistically promote mitotic chromosome condensation. Elife.

[bib38] Küey C., Larocque G., Clarke N.I., Royle S.J. (2019). Unintended perturbation of protein function using GFP nanobodies in human cells. J. Cell Sci..

[bib39] Lenne P.-F., Wawrezinieck L., Conchonaud F., Wurtz O., Boned A., Guo X.-J., Rigneault H., He H.-T., Marguet D. (2006). Dynamic molecular confinement in the plasma membrane by microdomains and the cytoskeleton meshwork. EMBO J..

[bib40] Leslie M. (2018). Small but mighty. Science.

[bib41] Lippincott-Schwartz J., Patterson G.H. (2009). Photoactivatable fluorescent proteins for diffraction-limited and super-resolution imaging. Trends Cell Biol.

[bib42] Maidorn M., Olichon A., Rizzoli S.O., Opazo F. (2019). Nanobodies reveal an extra-synaptic population of SNAP-25 and Syntaxin 1A in hippocampal neurons. MAbs.

[bib43] Maier J., Traenkle B., Rothbauer U. (2015). Real-time analysis of epithelial-mesenchymal transition using fluorescent single-domain antibodies. Sci. Rep..

[bib44] Mikhaylova M., Cloin B.M.C., Finan K., Van Den Berg R., Teeuw J., Kijanka M.M., Sokolowski M., Katrukha E.A., Maidorn M., Opazo F. (2015). Resolving bundled microtubules using anti-tubulin nanobodies. Nat. Commun..

[bib45] Niwa H., Inouye S., Hirano T., Matsuno T., Kojima S., Kubota M., Ohashi M., Tsuji F.I. (1996). Chemical nature of the light emitter of the Aequorea green fluorescent protein. Proc. Natl. Acad. Sci. U S A.

[bib46] Özhan G., Sezgin E., Wehner D., Pfister A.S., Kühl S.J., Kagermeier-Schenk B., Kühl M., Schwille P., Weidinger G. (2013). Lypd6 enhances Wnt/β-Catenin signaling by promoting Lrp6 phosphorylation in raft plasma membrane domains. Dev. Cell.

[bib47] Pereira P.M., Albrecht D., Culley S., Jacobs C., Marsh M., Mercer J., Henriques R. (2019). Fix Your membrane receptor imaging: actin cytoskeleton and CD4 membrane organization disruption by chemical fixation. Front. Immunol..

[bib48] Della Pia E.A., Martinez K.L. (2015). Single domain antibodies as a powerful tool for high quality surface plasmon resonance studies. PLoS One.

[bib49] Pinkwart K., Schneider F., Lukoseviciute M., Sauka-Spengler T., Lyman E., Eggeling C., Sezgin E. (2019). Nanoscale dynamics of cholesterol in the cell membrane. J. Biol. Chem..

[bib50] Platonova E., Winterflood C.M., Junemann A., Albrecht D., Faix J., Ewers H. (2015). Single-molecule microscopy of molecules tagged with GFP or RFP derivatives in mammalian cells using nanobody binders. Methods.

[bib51] Pleiner T., Bates M., Trakhanov S., Lee C.T., Schliep J.E., Chug H., Böhning M., Stark H., Urlaub H., Görlich D. (2015). Nanobodies: site-specific labeling for super-resolution imaging, rapid epitope- mapping and native protein complex isolation. Elife.

[bib52] Pleiner T., Bates M., Görlich D. (2018). A toolbox of anti-mouse and anti-rabbit IgG secondary nanobodies. J. Cell Biol..

[bib54] Rankin B.R., Moneron G., Wurm C.A., Nelson J.C., Walter A., Schwarzer D., Schroeder J., Colón-Ramos D.A., Hell S.W. (2011). Nanoscopy in a living multicellular organism expressing GFP. Biophys. J..

[bib55] Reina F., Galiani S., Shrestha D., Sezgin E., De Wit G., Cole D., Christoffer Lagerholm B., Kukura P., Eggeling C. (2018). Complementary studies of lipid membrane dynamics using iSCAT and super-resolved fluorescence correlation spectroscopy. J. Phys. D. Appl. Phys..

[bib56] Ries J., Kaplan C., Platonova E., Eghlidi H., Ewers H. (2012). A simple, versatile method for GFP-based super-resolution microscopy via nanobodies. Nat. Methods.

[bib58] Saffarian S., Elson E.L. (2003). Statistical analysis of fluorescence correlation spectroscopy: the standard deviation and bias. Biophys. J..

[bib59] Saffman P.G., Delbrück M. (1975). Brownian motion in biological membranes. Proc. Natl. Acad. Sci. U S A.

[bib60] Saha S., Lee I.-H., Polley A., Groves J.T., Rao M., Mayor S. (2015). Diffusion of GPI-anchored proteins is influenced by the activity of dynamic cortical actin. Mol. Biol. Cell.

[bib61] Sahl S.J., Hell S.W., Jakobs S. (2017). Fluorescence nanoscopy in cell biology. Nat. Rev. Mol. Cell Biol..

[bib63] Schneider F., Waithe D., Clausen M.P., Galiani S., Koller T., Ozhan G., Eggeling C., Sezgin E. (2017). Diffusion of lipids and GPI-anchored proteins in actin-free plasma membrane vesicles measured by STED-FCS. Mol. Biol. Cell.

[bib64] Schneider F., Waithe D., Lagerholm B.C., Shrestha D., Sezgin E., Eggeling C., Fritzsche M. (2018). Statistical analysis of scanning fluorescence correlation spectroscopy data differentiates free from hindered diffusion. ACS Nano.

[bib65] Schneider F., Hernandez-Varas P., Lagerholm C.B., Shrestha D., Sezgin E., Roberti J.M., Ossato G., Hecht F., Eggeling C., Urbančič I. (2020). High photon count rates improve the quality of super-resolution fluorescence fluctuation spectroscopy. J. Phys. D. Appl. Phys..

[bib66] Schwille P., Meyer-Almes F.J., Rigler R. (1997). Dual-color fluorescence cross-correlation spectroscopy for multicomponent diffusional analysis in solution. Biophys. J..

[bib67] Sograte-Idrissi S., Oleksiievets N., Isbaner S., Eggert-Martinez M., Enderlein J., Tsukanov R., Opazo F. (2019). Nanobody detection of standard fluorescent proteins enables multi-target DNA-PAINT with high resolution and minimal displacement errors. Cells.

[bib68] Sograte-Idrissi S., Schlichthaerle T., Duque-Afonso C.J., Alevra M., Strauss S., Moser T., Jungmann R., Rizzoli S.O., Opazo F. (2020). Circumvention of common labelling artefacts using secondary nanobodies. Nanoscale.

[bib69] Stanly T.A., Fritzsche M., Banerji S., García E., Bernardino de la Serna J., Jackson D.G., Eggeling C. (2016). Critical importance of appropriate fixation conditions for faithful imaging of receptor microclusters. Biol. Open.

[bib70] Stepanenko O., Verkhusha V., Kuznetsova I., Uversky V., Turoverov K. (2008). Fluorescent proteins as biomarkers and biosensors: throwing color lights on molecular and cellular processes. Curr. Protein Pept. Sci..

[bib71] Sun Y., Day R.N., Periasamy A. (2011). Investigating protein-protein interactions in living cells using fluorescence lifetime imaging microscopy. Nat. Protoc..

[bib72] Suzuki K.G.N., Kasai R.S., Hirosawa K.M., Nemoto Y.L., Ishibashi M., Miwa Y., Fujiwara T.K., Kusumi A. (2012). Transient GPI-anchored protein homodimers are units for raft organization and function. Nat. Chem. Biol..

[bib73] Suzuki T., Arai S., Takeuchi M., Sakurai C., Ebana H., Higashi T., Hashimoto H., Hatsuzawa K., Wada I. (2012). Development of cysteine-free fluorescent proteins for the oxidative environment. PLoS One.

[bib75] Traub L.M. (2019). A nanobody-based molecular toolkit provides new mechanistic insight into clathrin-coat initiation. Elife.

[bib76] Tsien R.Y. (1998). The green fluorescent protein. Annu. Rev. Biochem..

[bib77] Veerapathiran S., Wohland T. (2017). The imaging FCS diffusion law in the presence of multiple diffusive modes. Methods.

[bib79] Waithe D., Schneider F., Chojnacki J., Clausen M.P., Shrestha D., de la Serna J.B., Eggeling C. (2017). Optimized processing and analysis of conventional confocal microscopy generated scanning FCS data. Methods.

[bib80] Wang X., Song K., Li Y., Tang L., Deng X. (2019). Single-molecule imaging and computational microscopy approaches clarify the mechanism of the dimerization and membrane interactions of green fluorescent protein. Int. J. Mol. Sci..

[bib81] Weiß K., Neef A., Van Q., Kramer S., Gregor I., Enderlein J. (2013). Quantifying the diffusion of membrane proteins and peptides in black lipid membranes with 2-focus fluorescence correlation spectroscopy. Biophys. J..

[bib83] De Wit G., Danial J.S.H., Kukura P., Wallace M.I. (2015). Dynamic label-free imaging of lipid nanodomains. Proc. Natl. Acad. Sci. U S A.

[bib84] Zacharias D.A., Violin J.D., Newton A.C., Tsien R.Y. (2002). Partitioning of lipid-modified monomeric GFPs into membrane microdomains of live cells. Science.

