# Supplementary material for: Influence of nanobody binding on fluorescence emission, mobility, and organization of GFP-tagged proteins
Source: iScience. 2020 Dec 4;24(1):101891. doi: 10.1016/j.isci.2020.101891 (PMC7753935; doi:10.1016/j.isci.2020.101891)
Supplement: Document S1. Transparent methods and Figures S1–S17 [file mmc1.pdf]

iScience, Volume *24*

Supplemental Information

Influence of nanobody binding on fluorescence emission, mobility, and organization of GFP-tagged proteins

Falk Schneider, Taras Sych, Christian Eggeling, and Erdinc Sezgin

Transparent Methods

Cell culture & labelling

PtK2 cells were cultured at 37 °C, 5 % CO_2, in DMEM (Sigma Aldrich) supplemented with 16% FBS (Sigma Aldrich). For microscopy the cells were seeded on 25 mm diameter glass coverslips (#1.5 thickness). Transfections of GPI-EGFP (kind gift from Kai Simon's lab) and GFP-LYPD6 (Özhan et al., 2013) were performed with Lipofectamine 3000 (Thermo Fisher) according to the manufacturer's protocol.

The protein sequences of the specific fluorescent protein variants used in this study are:

(GPI-)EGFP

MVSKGEELFTGVVPILVELDGDVNGHKFSVSGEGEGDATYGKLTLKFICTTGKLPVPWPTLV
TTLTYGVQCFSRYPDHMKQHDFFKSAMPEGYVQERTIFFKDDGNYKTRAEVKFEGDTLVNR
IELKGIDFKEDGNILGHKLEYNYNSHNVYIMADKQKNGIKVNFKIRHNIEDGSVQLADHYQQ
NTPIGDGPVLLPDNHYLSTQSALSKDPNEKRDHMVLLEFVTAAGITLGMDELYK

(LYPD6-)GFP

MKRHDFFKSAMPEGYVQERTIFFKDDGNYKTRAEVKFEGDTLVNRIELKGIDFKEDGNILGH
KLEYNYNSHNVYIMADKQKNGIKANFKTRHNIEDGGVQLADHYQQNTPIGDDPVLLPDNHY
LSTQSALSKDPNEKRDHMVLLEFVTAAGITHGMDELYKP

While imaging cells at 37 °C, they were incubated with unlabelled (GFP-binding protein, Chromotek) and Abberior 635P conjugated anti-GFP nanobody (GFP-booster, Chromotek). All experiments were performed in L15 imaging medium (Sigma Aldrich) and 1 μL of the nanobody added to 1 mL and well mixed. For all measured conditions it can be assumed that the nanobody was added well in excess to saturate the fluorescent proteins and maximise binding.

GUVs

GUVs were prepared using electro-formation as described in (Jenkins et al., 2019). Lipid mixture (1mg/mL DOPC:DGS-Ni-NTA (both obtained from Avanti Polar Lipids) 98:2 molar ratio) were spread on platinum wire and dipped into 300 mM sucrose. GUVs formed after exposure to an AC filed of 2 V and 10 Hz for 1 h followed by 2 V 2 Hz for another 30 minutes. Different amounts (5 μg/ml down to 0.01 μg/ml) of His-tagged GFP (Sino Biological) and EGFP (OriGene) was incubated for 20 minutes with the vesicles and then carefully washed before imaging and FCS was performed. Lack of excess GFP was confirmed with FCS in the GUV background solution (Supplementary Figure S16). The nanobody was directly added to the imaging chamber containing GUVs in PBS (total volume of 300 μL).

Bead supported lipid bilayers

Bead supported lipid bilayers (BSLBs) were prepared as described previously (Beckers et al., 2020). Briefly, BSLBs were prepared from spontaneous fusion of liposomes of 1 mg/ml lipid stock with 5 μm silica beads obtained from Bangs Laboratories. Liposomes were prepared by tip sonication (Beckers et al., 2020). Silica beads were washed twice with 1 ml PBS. Beads were mixed with liposomes (1:7) and then shaken for 20 minutes at 1500 rpm to form BSLBs. BSLBs were washed twice with PBS. BSLBs are uniform in size and can be produced in large numbers easily (unlike GUVs), thus are convenient tools for quantitative membrane biology. We used them to quantify the concentration dependence of intensity increase upon Nb binding.

Confocal microscopy & FCS

Confocal microscopy and FCS were performed on Zeiss780 and Zeiss880 LSMs both equipped with an Argon laser for fluorescence excitation. All sFCS and most imaging has been performed in photon counting mode using Channel S. To excite the labelled nanobody the HeNe 633 excitation has been employed. For single colour FCS and imaging a 488 dichroic mirror and for two-colour imaging a 488/561/633 MBS was used. The fluorescence was

collected between 500 nm and 600 nm for the green channel and between 640 nm and 695 nm for the red channel. Laser powers were between 1 and 5 µW and kept below saturation to avoid artefacts in FCS.

The images were processed using FIJI (Rueden et al., 2017; Schindelin et al., 2012). The plasma membranes of each cell was segmented out using the polygon selection tool. Similar sized regions of interest were generated for the background. The average intensities over time of the membrane areas for all segmented cells were extracted using the ImageJ built-in function "z-profile" (note that z does only refer to the third image dimension and is in this case time).

The quantitative analysis of the microscopy images of BSLBs was performed using the new release of the Fiji-based macro GUV-AP1 (Sych et al., 2019). Briefly, individual BSLBs were detected and the mean fluorescence intensity of GFP at the rim of each BSLB was quantified (Supplementary Figure S17). The source code of the macro is available at https://github.com/taras-sych/GUV-AP/tree/GUV-AP-v-3.0.

Point-FCS measurements were performed using Zeiss' internal FCS routine. Measurements were between 10 and 15 seconds long. The objective's correction collar was adjusted and the pinhole aligned measuring the diffusion of Alexa Fluor 488 in water. FCS measurements were saved as .fcs files for fitting. The same procedure was followed for cross-correlation (FCCS) measurements but additionally using a cross-correlation positive control (Bodipy and Alexa647 labelled HDL particles (Plochberger et al., 2017)) to ensure optical alignment.

sFCS measurements were performed as xt scans. 52 pixels were acquired for 10^5 lines at about 2000 Hz yielding a pixel dwell time of 3.94 µs (overall resulting in a total acquisition time of about 47 seconds). The data were saved as .lsm5 file and externally correlated using the FoCuS_scan software package (Waithe et al., 2017). To correct for photobleaching, the first seconds were cropped off and a local averaging bleaching-correction applied as described in

(Waithe et al., 2017). sFCCS measurements were performed in a similar manner using the described acquisition settings in conjunction with the optical set-up for two-colour imaging as described above. As a positive control for sFCCS, we used a sparse sample of DOPC (1,2-dioleoyl-sn-glycero-3-phosphocholine), Avanti Polar Lipids) vesicles doped 1:50,000 with DiO (Invitrogen) and 1:10,000 with AbberiorSTAR-Red-PEG-Cholesterol (Abberior). The sample was prepared by mixing the lipid and dyes in ethanol, drying the mixture, and re-suspending the lipid film by vortexing and sonication (5 minutes and 30 minutes, respectively) in water. Measurements were performed in PBS.

Point and sFCS data were fitted in FoCuS (Waithe et al., 2016, 2017). Point-FCS data showed a contribution of a triplet component (40 µs for (E)GFP (Schneider et al., 2020)), a fast (probably cytoplasmic) component, with transit times around 0.1 ms, and a slower transit time which was attributed to the diffusion in the membrane. Thus, pFCS data were fitted with a two component diffusion model including a triplet state (Widengren et al., 1995) in the lag time regime from 0.001 ms to 1000 ms. sFCS acquisitions miss the fast dynamics and were fitted with a single component 2D diffusion model (Schneider et al., 2018). Fitting in FoCuS is performed using a Levenberg-Marquard non-linear least square optimisation. The fitted parameters including the cpms (determined from fitted amplitude and the average count rate) were exported as Excel sheets and post processed with Matlab or GraphPad Prism 8.

Some data on mobility were acquired cell by cell to account for the inherent biological heterogeneity. In these cases, only the ratio of the transit times, number of molecules or counts per molecule before and after addition of the nanobody are reported (After/Before). Statistical tests were performed in GraphPad Prism 8. We employed the Wilcoxon sign-rank non-parametric tests with hypothetical median values of 1 for the data presented as ratios and we used the Kolmogorov–Smirnov non-parametric test for all other data.

Lifetime measurements

Life time imaging was performed on a Microtime 200 (PicoQuant) equipped with a FlimBee galvo scanner. Fluorescence was excited with a 488 nm diode laser (PicoQuant) and focused onto the sample with an Olympus UPlanSApo 60 x water-immersion objective. Images were acquired as 50 by 50 μm^2 (512 by 512 pixel) for collecting the fluorescence for 60 s at low excitation power (<1 μW) to avoid too high count rates and distortion of the TCSPC data (Isbaner et al., 2016). The overall TCSPC curves were used for lifetime fitting. First all data were fitted with a 2 component tail fit. The second component fluctuated around 4.1 ns and was fixed to this value for a subsequent round of tail-fitting. We report the amplitude weighted lifetimes of GPI-eGFP and GFP-LYPD6 in the plasma membrane of living PtK2 cells.

The same set-up was used to measure the fluorescent lifetimes of GFP-His and EGFP-His in solution. These data were acquired as point scans.

SPR

We immobilised the nanobodies (either fluorescently labelled or unlabelled) by amine coupling to a CM5 chip (with an RFPNB in the reference channel) then injected GFP as the analyte in a kinetic analysis (a single injection at 87 nM, using curve fitting in the BiaEvaluation software to measure on and off rates from which the K_D is calculated).

Spectra

All spectral measurements were performed using a CLARIO STAR plate reader (BMG LABTECH). His-tagged GFP and EGFP were measured at varying concentrations from 2.5 μg/mL down to 0.01 μg/mL in PBS (pH 7.4) in glass bottom 96-well plates (Porvair Sciences) which were prior to the measurements coated with BSA to prevent sticking of the fluorescent proteins to the glass. The nanobody was added as supplied from Chromotek and used in ~ 8-fold excess. All spectra are averages of multiple wells. Excitation scan were performed with a

readout at 510 nm and emission scans were performed with excitation at 405 nm or 488 nm. We choose a spectral resolution of 1 nm and used 200 flashes per wavelength for averaging.

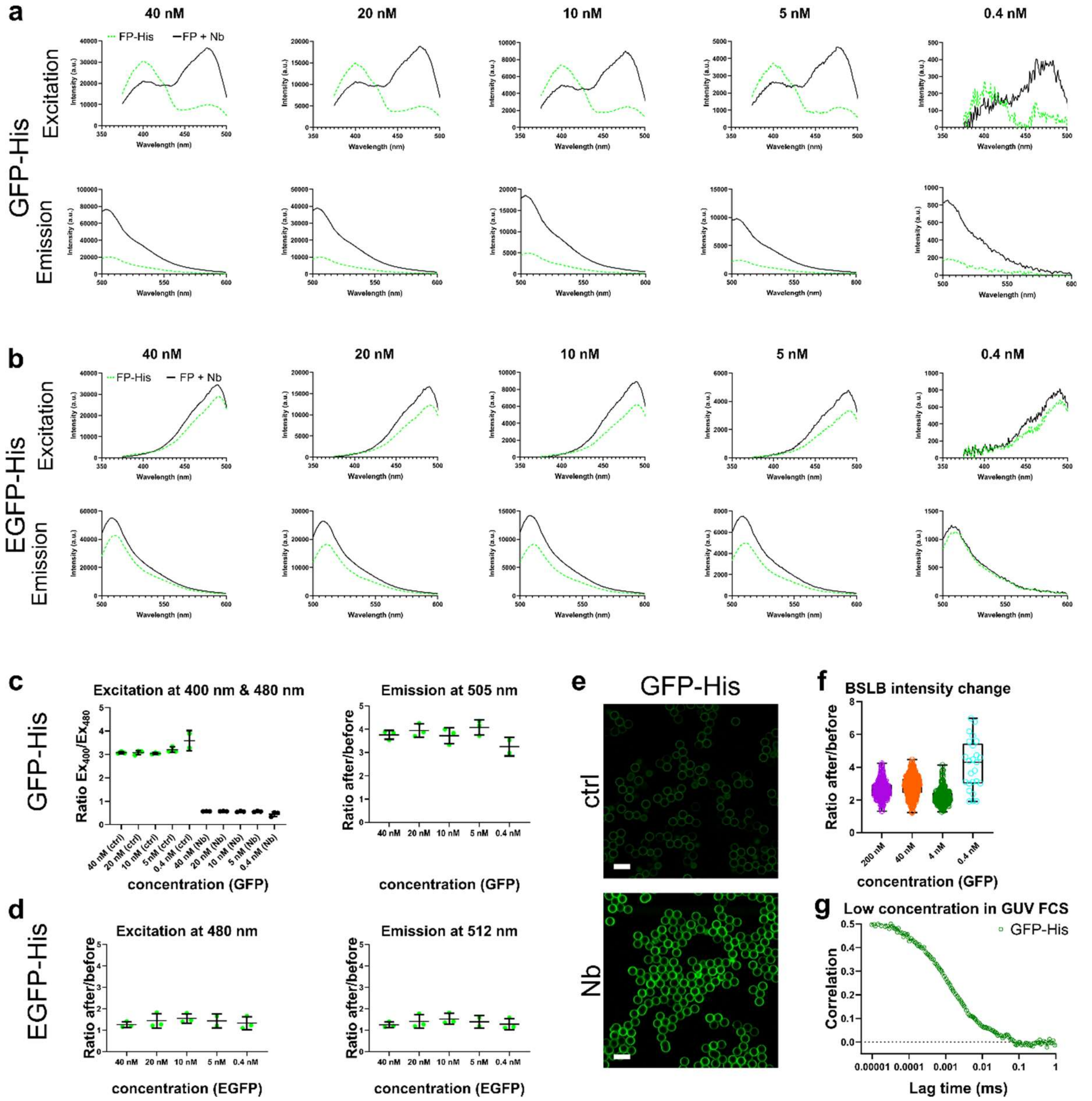

Supplementary Figure S1: Nb induced changes in fluorescence properties of EGFP-His and GFP-His at different concentrations in solution or on BSLBs, Related to Figure 1. Excitation (top) and emission (bottom) spectra of (a) GFP-His and (b) EGFP-His in PBS (pH 7.4) at different concentrations (as indicated) without nanobody (FP, dashed green lines) and with nanobody (FP+Nb, black solid lines) in 8-fold excess. Shown spectra are averages of 3 measurements. c) Ratio of excitation peaks at 400 nm and 480 nm as well as emission at 500 nm for GFP-His in solution and treated with nanobody. d) Excitation at 480 nm and emission at 512 nm enhancement by nanobody binding given as ratio after/before treatment. e) Confocal images of BSLBs containing 98 mol% POPC and 2 mol% DGS-Ni-NTA and labelled with GFP-His (ctrl, top) and treated with nanobody (Nb, bottom). f) Quantification of intensity increase as ratio of after/before nanobody treatment at different concentrations of GFP-His. Every dot represents the radial average intensity from one BSLB. Scale bar 10 µm. g) FCS auto correlation curve of GFP-His indicating low concentrations used in the experiments (amplitude of 0.5 ~ 2 molecules in focus).

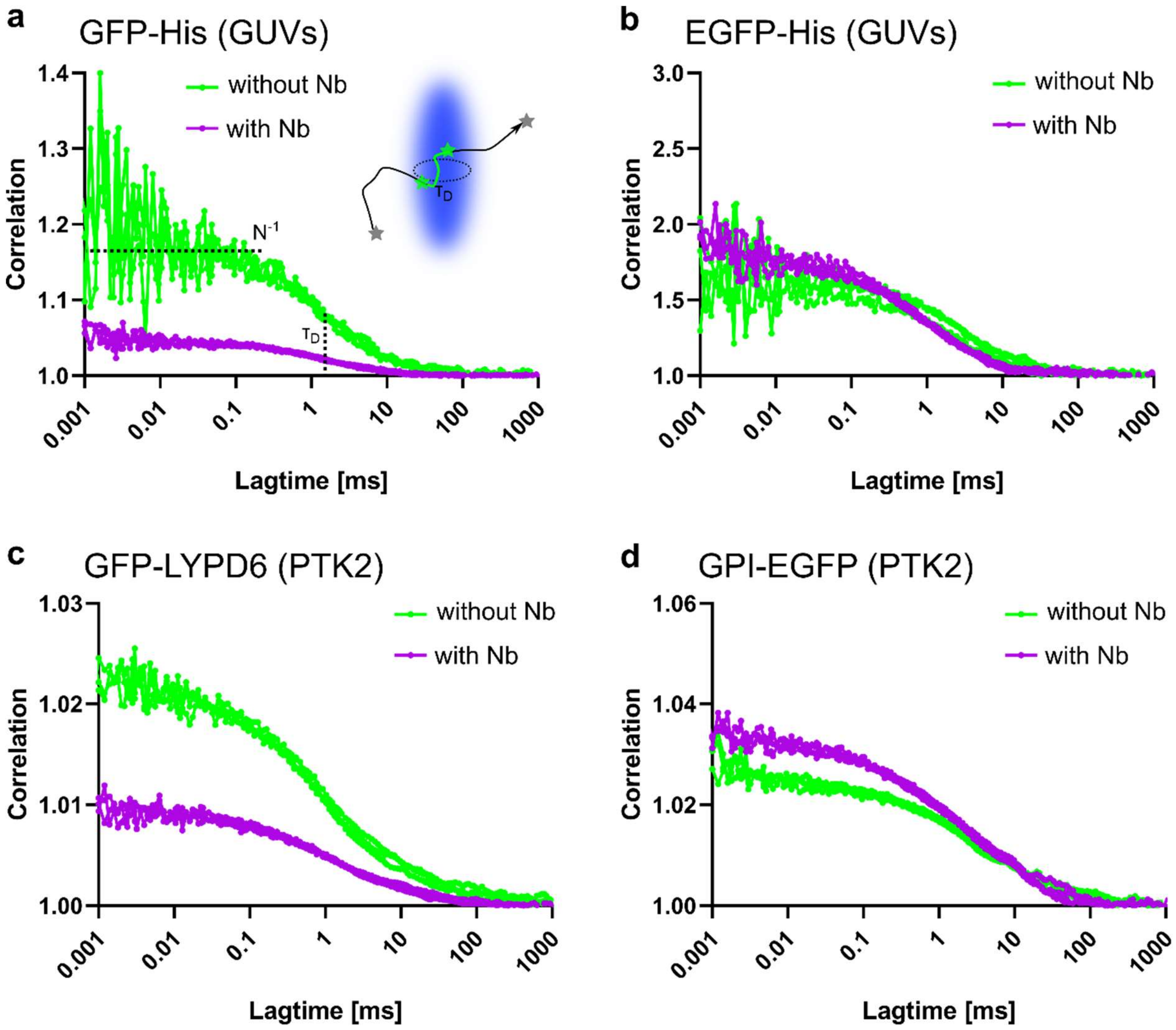

Supplementary Figure S2: Representative point FCS data, Related to Figure 2. GFP (left panels) and EGFP (right panels) diffusing on different membrane systems, GUVs (98 mol% DOPC and 2 mol% DGS-Ni-NTA) decorated with GFP-His (a) and EGFP-His (b), and plasma membrane of live PtK2 cells for GFP-LYPD6 (c) and GPI-EGFP (d) with (magenta) and without (green) addition of unlabelled Nb; three representative curves are shown for each condition. Inset of a) schematic of FCS measurements for the determination of the average molecular brightness or count-rate per molecule (cpm), the average number of fluorescing molecules in the observation spot (N), and the average transit time τ_D through the observation spot (blue) as measure of the molecular mobility.

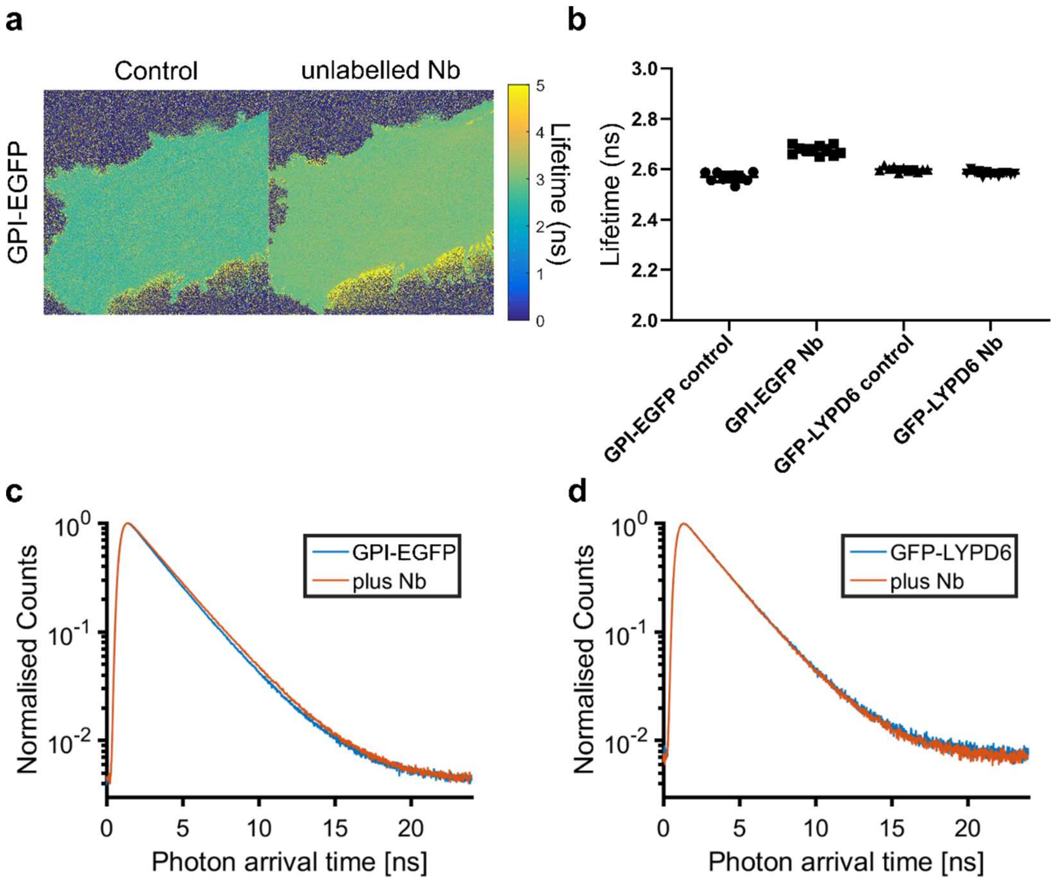

Supplementary Figure S3: Fluorescence lifetime imaging of GPI-EGFP and GFP-LYPD6, Related to Figure 3. a) Representative lifetime images of the basal plasma membrane of live PtK2 cells transfected with GPI-EGFP (left) and treated with unlabelled Nb (right). Image size 50x50 μm^2. b) Average values of fluorescence lifetimes of the same fluorescent proteins as determined from fitting the respective TCSPC-based fluorescence decays averaged over the whole image (c,d). Note that amplitude weighted lifetimes from a bi-exponential tail fit are given as lifetimes for both GPI-EGFP and GFP-LYPD6.

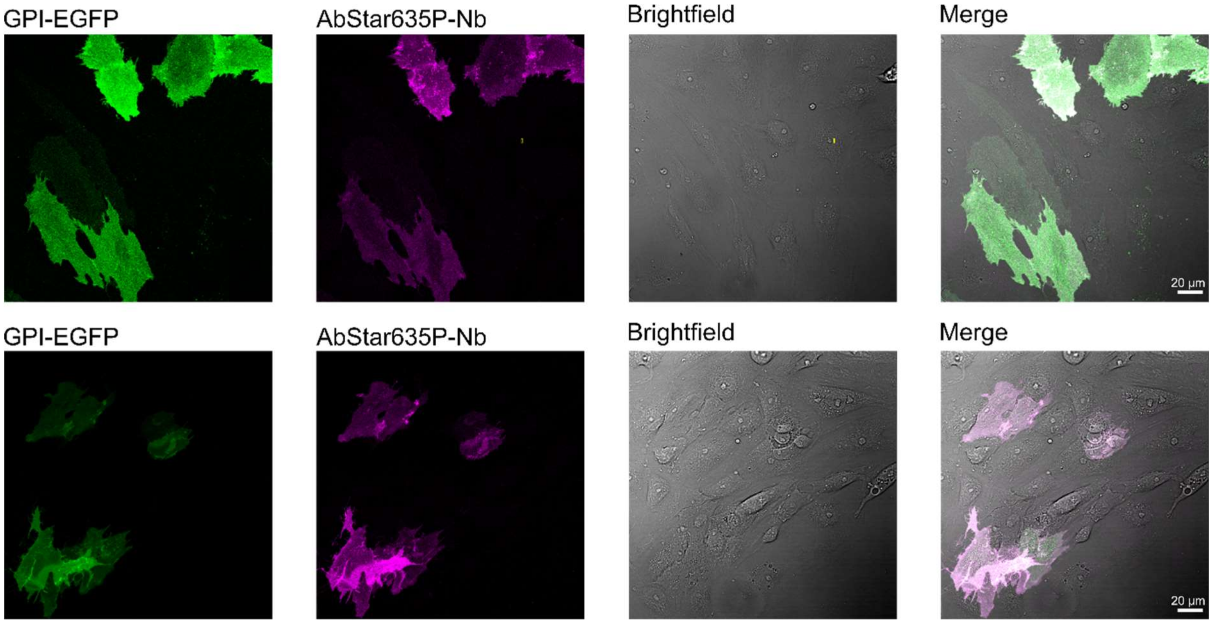

Supplementary Figure S4: Specific binding of the labelled nanobody (AbStar635P-Nb) to transfected cells, Related to Figure 4. Representative confocal images of PtK2 cells expressing GPI-EGFP and additionally stained with Abberior Star 635P-labelled nanobodies (AbStar635P-Nb): EGFP (left), AbStar635P-Nb (middle left), brightfield transmission (middle right) and merged (right) observation channels. Scale bars 20 µm.

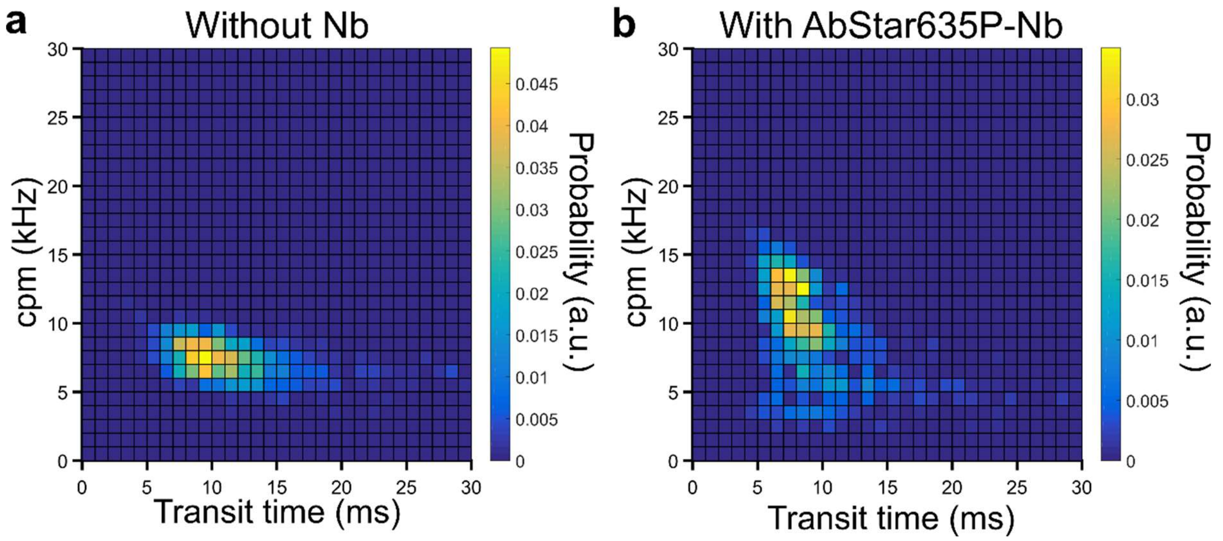

Supplementary Figure S5: Also labelled Nb causes shifts in mobility and brightness, Related to Figure 4. Two-dimensional pair value histograms (bi-variate histograms) of transit times and cpms from sFCS experiments on GPI-EGFP on live PtK2 cells without (a) and with a labelled Nb (AbStar635P, b). The observed shifts are similar to the unlabelled Nb in Figure 4d,f.

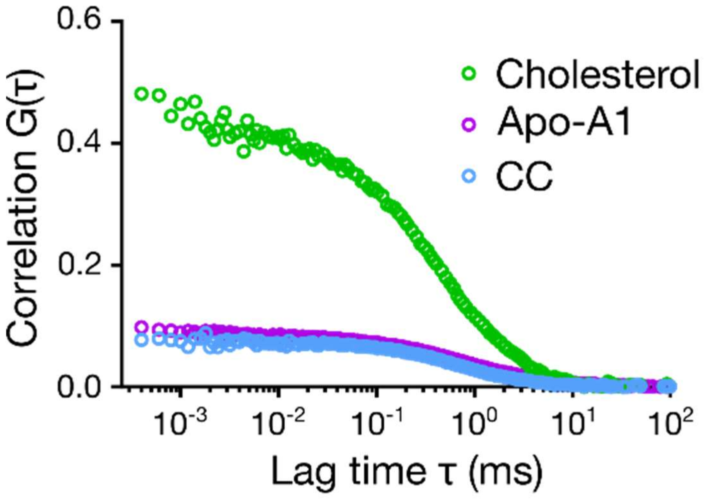

Supplementary Figure S6: FCCS positive control, Related to Figure 6. Representative FCCS data (autocorrelation (green and magenta) and cross-correlation (CC, blue) curves) for HDL particles labelled with Bodipy-cholesterol and ApoA1-Alexa647, demonstrating the capability of the set-up to obtain almost 100% cross-correlation for a perfect sample.

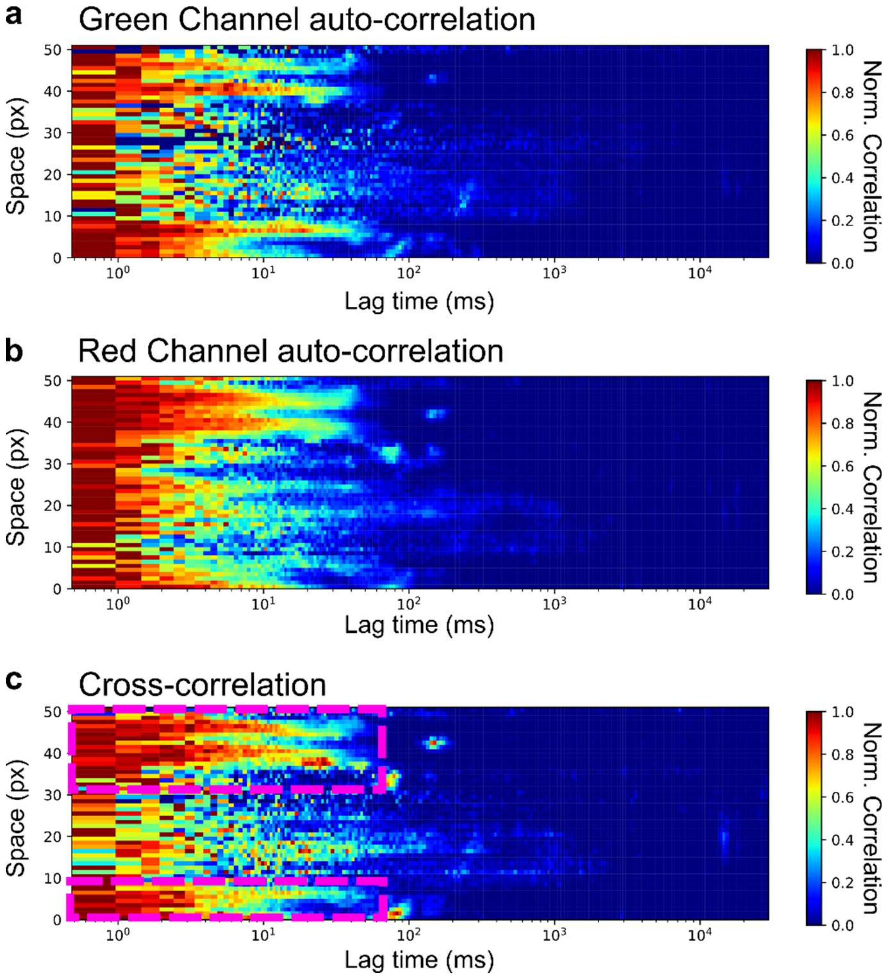

Supplementary Figure S7: sFCCS positive control, Related to Figure 6. Exemplary sFCCS measurement of a sparse vesicle solution. Vesicles were made of DOPC doped with DiO and AbberiorSTAR-Red-PEG-Cholesterol. Normalised auto-correlation for the green (a, Fast-DiO) and red channel (b, AbberiorSTAR-Red), and cross-correlation of both (c). The dashed magenta boxes in c) indicate positions of clear cross-correlation resembling the respective auto-correlations and revealing co-diffusion.

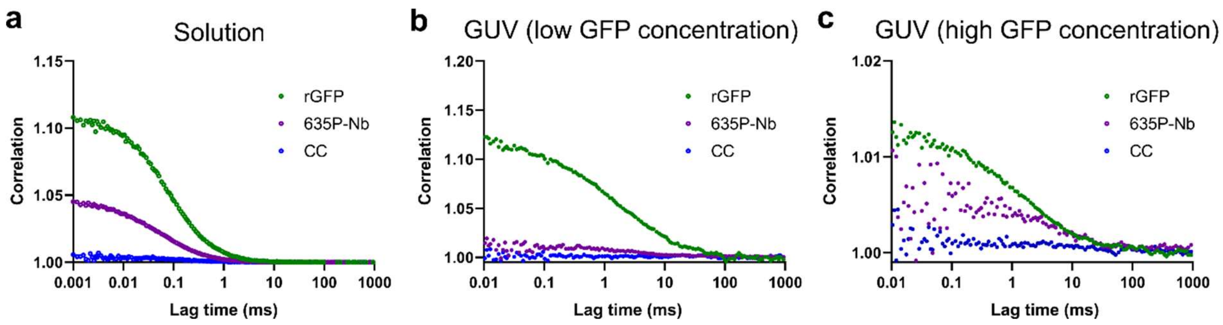

Supplementary Figure S8: Lack of co-diffusion (cross-correlation, CC, blue) between recombinant GFP (rGFP, green) and Nb-AbSt635P (magenta) in solution and model membranes, Related to Figure 6. Data shown are average of three curves from point FCCS acquisitions in (a) PBS or in GUVs (98 mol% POPC and 2 mol% DGS-Ni-NTA) with (b) low (0.4 nM) or (c) high (4 nM) concentration of His-tagged GFP.

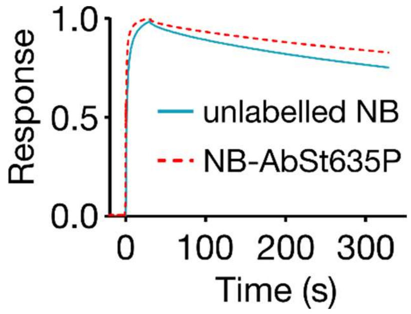

Supplementary Figure S9: Nanobody and GFP interaction kinetics, Related to Figure 6. Surface plasmon resonance (SPR) plots showing the strong (and fast) binding of His-GFP to immobilized unlabeled (blue) or Abberior Star 635P tagged Nb (dashed orange) suggesting that the complex should be stable over the course of the experiments (k_{off}= 5.554 e^{-4} s^{-1} and k_d=3.8 e^{-11} M).

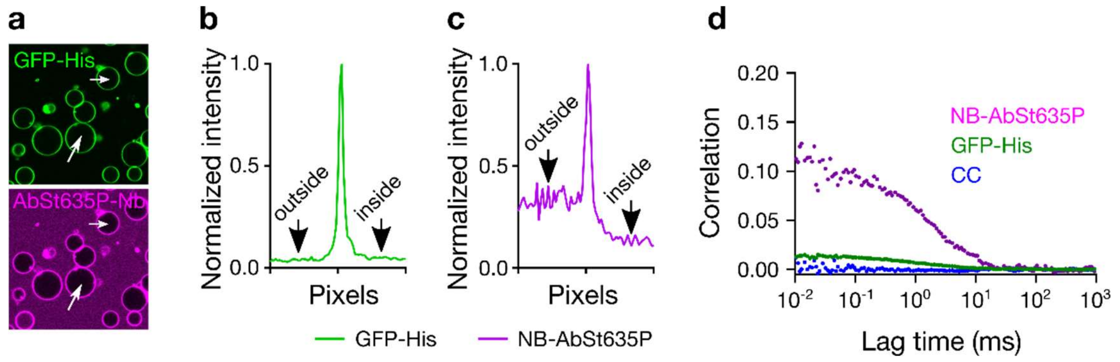

Supplementary Figure S10: FCCS measurements at excess Nb, Related to Figure 6. a) Confocal images of GUVs decorated with His-GFP and incubated with AbStar635P-Nb (80 µm x 80 µm). b, c) The line profile of the arrows shown in panel a for b) GFP and c) AbStar635P-Nb. The image and the line profile show excess Nb in the solution. d) Autocorrelation and cross-correlation for GFP and AbStar635P-Nb. No cross-correlation is observed when performing point FCCS measurements on the GUVs.

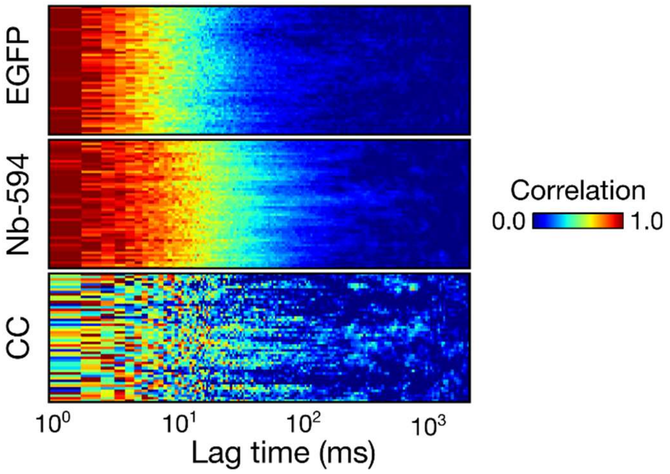

Supplementary Figure S11: No influence of the label on the nanobody, Related to Figure 6. Representative cross-correlation is also absent using a nanobody labelled with Atto594. sFCCS measurement on GPI-EGFP transfected PtK2 cells additionally tagged with a labelled nanobody (labelled with Atto594). The nanobody channel resembles the familiar slow-down but only neglectable cross-correlation (CC) can be observed.

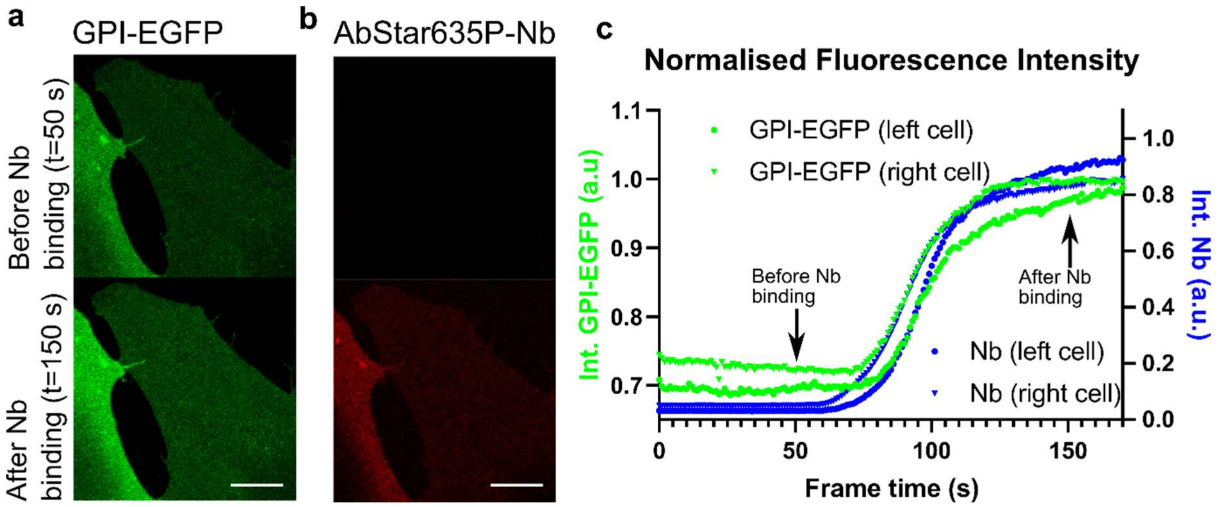

Supplementary Figure S12: Changes in fluorescence intensity upon addition of AbStar635P-Nb to live PtK2 cells, Related to Figure 6. a,b) Representative confocal images of the basal plasma membrane of PtK2 cells expressing GPI-EGFP (green) before and after addition of AbStar635P labelled Nb (red) taken from an image stack at t=50s and t=150s after Nb addition. c) Extracted and normalized fluorescence intensity time traces for both cells in a,b.

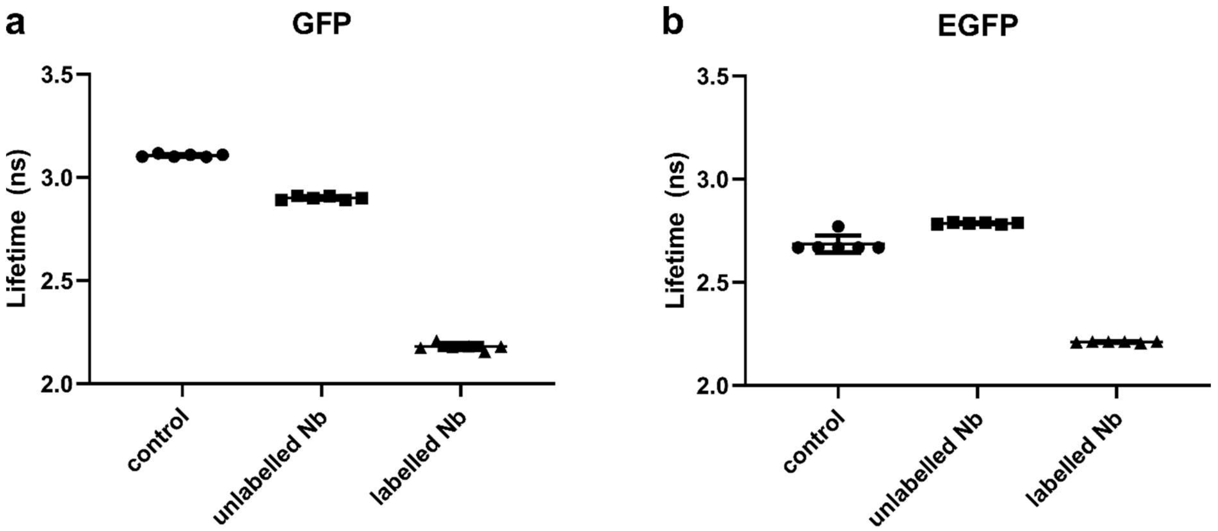

Supplementary Figure S13: Fluorescence lifetimes in solution, Related to Figure 6. Fluorescence lifetimes as determined from TCSPC measurements in PBS (pH 7.4) excited with 488 nm for a) GFP and b) EGFP as well as in presence of excess unlabelled or Abberior STAR 635P labelled Nb. Note that for EGFP (control and labelled Nb) a bi-exponential fit had to be employed and amplitude weighted lifetimes are given. For all other data a mono-exponential tail-fit was sufficient.

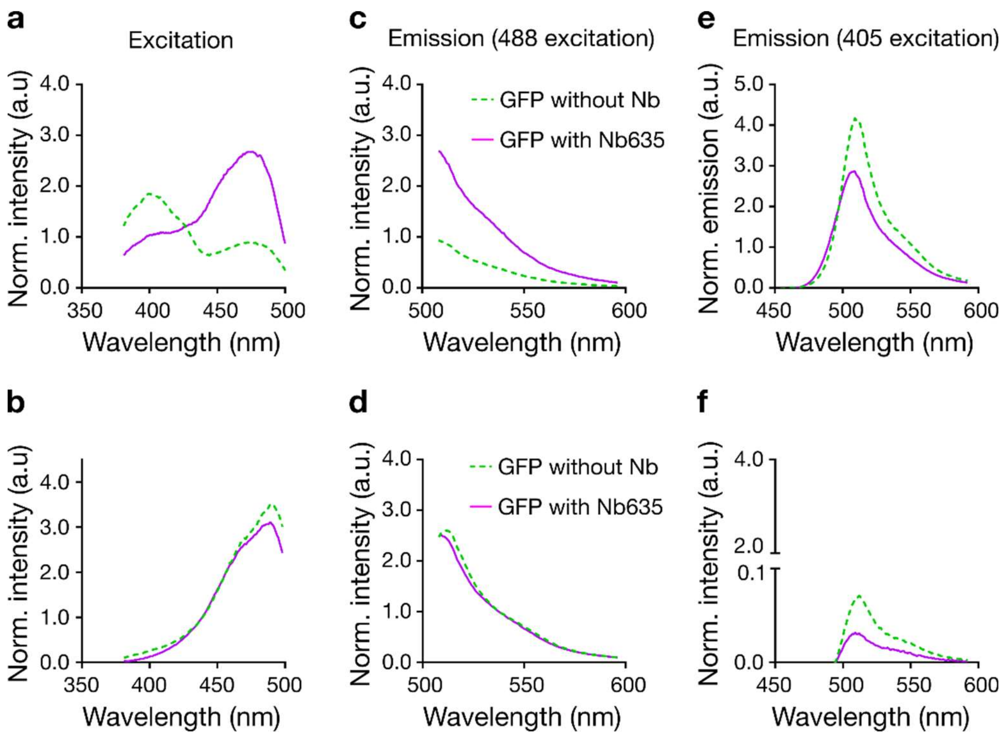

Supplementary Figure S14: Change in excitation and emission spectra of recombinant GFP and EGFP in solution upon addition of fluorescently labelled Nb, Related to Figure 6. Excitation spectra for fluorescence detection at 510 - 520 nm (a,b) and emission spectra following 488 nm (c,d) and 405 nm excitation (e, f) of GFP (a,c,e) and EGFP (b,d, f) without Nb (green dashed line) and with labelled AbStar635P-Nb (magenta solid lines). All spectra are averages of three measurements.

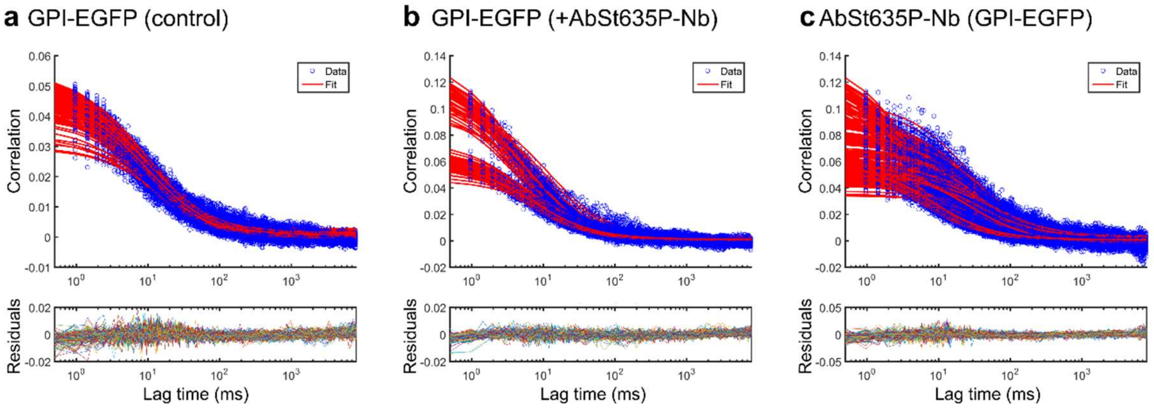

Supplementary Figure S15: **Fitting of sFCS data with a 1-component model, Related to Figure 4:** sFCS autocorrelation data (blue circles) and fitted curves (red lines) in the top panels and residuals in the bottom panels. For GPI-EGFP (a), GPI-EGFP after addition of Abberior STAR 635P labelled nanobody (AbSt635P-Nb) (b), and the nanobody itself (red channel) (c) a single component fit represent the data well. Shown are ~150 curves for each condition (3 sFCS measurements from different cells). The data in (b) and (c) were acquired simultaneously.

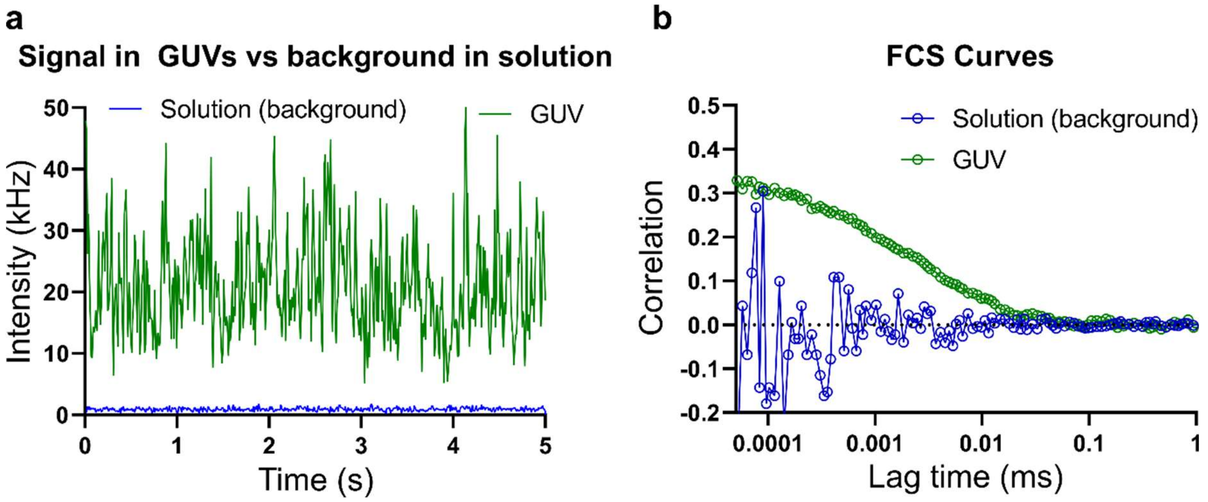

Supplementary Figure S16: **Lack of free GFP in the GUV imaging chamber, Related to Figure 2.** a) Intensity traces for EGFP-His diffusing on a GUV (green, measurement on the GUV membrane) and the background (blue, measurement in solution far away from a GUV). b) FCS autocorrelation curves of the intensity traces in a indicating only noise (no freely diffusing molecules) in the solution (blue) and 2D diffusion at the membrane (green).

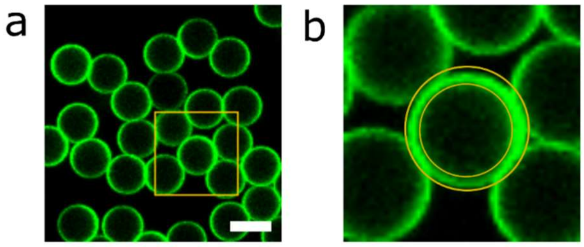

Figure S17. Processing BSLB images, Related to Figure 2. a) Representative image of BSLBs labeled with GFP. b) Mean fluorescence intensity of GFP at the rim of the BSLBs was quantified. The region of interest for fluorescence intensity quantification for each individual BSLB was constructed as indicated with the ring selection. Scale bar 5 µm.

Supplemental References

Beckers, D., Urbancic, D., and Sezgin, E. (2020). Impact of Nanoscale Hindrances on the Relationship between Lipid Packing and Diffusion in Model Membranes. J. Phys. Chem. B *124*, 1487–1494.

Isbaner, S., Karedla, N., Ruhlandt, D., Stein, S.C., Chizhik, A., Gregor, I., and Enderlein, J. (2016). Dead-time correction of fluorescence lifetime measurements and fluorescence lifetime imaging. Opt. Express *24*, 9429–9445.

Jenkins, E., Santos, A.M., O'Brien-Ball, C., Felce, J.H., Wilcock, M.J., Hatherley, D., Dustin, M.L., Davis, S.J., Eggeling, C., and Sezgin, E. (2019). Reconstitution of immune cell interactions in free-standing membranes. J. Cell Sci. *132*, jcs219709.

Özhan, G., Sezgin, E., Wehner, D., Pfister, A.S., Kühl, S.J., Kagermeier-Schenk, B., Kühl, M., Schwille, P., and Weidinger, G. (2013). Lypd6 Enhances Wnt/β-Catenin Signaling by Promoting Lrp6 Phosphorylation in Raft Plasma Membrane Domains. Dev. Cell *26*, 331–345.

Plochberger, B., Röhrl, C., Preiner, J., Rankl, C., Brameshuber, M., Madl, J., Bittman, R., Ros, R., Sezgin, E., Eggeling, C., et al. (2017). HDL particles incorporate into lipid bilayers – a combined AFM and single molecule fluorescence microscopy study. Sci. Rep. *7*, 15886.

Rueden, C.T., Schindelin, J., Hiner, M.C., DeZonia, B.E., Walter, A.E., Arena, E.T., and Eliceiri, K.W. (2017). ImageJ2: ImageJ for the next generation of scientific image data. BMC Bioinformatics *18*, 529.

Schindelin, J., Arganda-Carreras, I., Frise, E., Kaynig, V., Longair, M., Pietzsch, T., Preibisch, S., Rueden, C., Saalfeld, S., Schmid, B., et al. (2012). Fiji: an open-source platform for biological-image analysis. Nat. Methods *9*, 676–682.

Schneider, F., Waithe, D., Lagerholm, B.C., Shrestha, D., Sezgin, E., Eggeling, C., and Fritzsche, M. (2018). Statistical Analysis of Scanning Fluorescence Correlation Spectroscopy Data Differentiates Free from Hindered Diffusion. ACS Nano *12*, 8540–8546.

Schneider, F., Hernandez-Varas, P., Lagerholm, C.B., Shrestha, D., Sezgin, E., Roberti, J.M., Ossato, G., Hecht, F., Eggeling, C., and Urbančič, I. (2020). High photon count rates improve the quality of super-resolution fluorescence fluctuation spectroscopy. J. Phys. D. Appl. Phys. *53*, 164003.

Sych, T., Schubert, T., Vauchelles, R., Madl, J., Omidvar, R., Thuenauer, R., Richert, L., Mély, Y., and Römer, W. (2019). GUV-AP: multifunctional FIJI-based tool for quantitative image analysis of Giant Unilamellar Vesicles. Bioinformatics *35*, 2340–2342.

Waithe, D., Clausen, M.P., Sezgin, E., and Eggeling, C. (2016). FoCuS-point: software for STED fluorescence correlation and time-gated single photon counting. Bioinformatics *32*, 958–960.

Waithe, D., Schneider, F., Chojnacki, J., Clausen, M.P., Shrestha, D., de la Serna, J.B., and Eggeling, C. (2017). Optimized processing and analysis of conventional confocal microscopy generated scanning FCS data. Methods *140–141*, 62–73.

Widengren, J., Mets, U., and Rigler, R. (1995). Fluorescence correlation spectroscopy of triplet states in solution: a theoretical and experimental study. J. Phys. Chem. *99*, 13368–13379.
